# Supplementary material for: Connectome architecture shapes large-scale cortical alterations in schizophrenia: a worldwide ENIGMA study
Source: Mol Psychiatry. 2024 Feb 9;29(6):1869–81. doi: 10.1038/s41380-024-02442-7 (PMC11371638; doi:10.1038/s41380-024-02442-7)
Supplement: Supplementary file 1 — Supplemental Material [file 41380_2024_2442_MOESM1_ESM.pdf]

Connectome architecture shapes large-scale cortical alterations in schizophrenia: a worldwide ENIGMA study

Georgiadis et al.

Supplementary Methods and Results

Contents

- Table S1. Site-specific inclusion criteria
- Table S2. Site demographics
- Table S3. Sample image acquisition and image pre-processing details by cohort
- HCP MRI data and preprocessing
- Functional and structural connectivity matrix generation from HCP participants
- ComBat batch effect adjustment
- Table S4 ComBat batch effect adjustment confirmation
- Table S5. Cortical thickness differences between schizophrenia patients and healthy controls
- Table S6. Subcortical volume differences between schizophrenia patients and healthy controls
- Table S7. Schizophrenia cortical epicenter ranking
- Table S8. Schizophrenia subcortical epicenter ranking
- Tables S9. Divergent and convergent regions of different stages of schizophrenia
- Mega-analysis robustness and sensitivity analyses
  - Reproducibility across different centrality metrics
  - Table S10. Correlation matrix of centrality measures
  - Table S11. Hub vulnerability across centrality metrics
  - Reproducibility across HCP age-matched and age-divergent ENIGMA schizophrenia samples
  - Robustness and site-specific confirmation analysis of morphological alterations, hub vulnerability and disease epicenter models in SCZ
  - Table S13. Correlation between site-specific and mega-analytic cortical alteration maps of schizophrenia
  - Figure S1. Site-specific epicenter mapping
  - Table S14. Site-specific hub vulnerability analysis
  - Table S15. Site-specific epicenter map agreement with mega-analytical epicenter map
- Subject-level cortical abnormality modeling
- Figure S2. Individual-level network modeling analysis in schizophrenia
- Subject-level correlation of clinical variables to hub vulnerability and epicenters
- Table S16. Correlations between subject-level functional and structural hub vulnerability and clinical scores
- Table S17. Correlations between individual subject-level functional epicenters and clinical scores
- Table S18. Correlations between individual subject-level structural epicenters and clinical scores
- Subject-level robustness and sensitivity analysis
- Figure S3. Stability of correlation of hub vulnerability at the individual level to all clinical variables

- Figure S4 Stability of correlation of epicenter values at the individual level to PANSS positive symptoms
- Figure S5. Stability of correlation of epicenter values at the individual level to PANSS negative symptoms
- Figure S6. Stability of correlation of epicenter values at the individual level to PANSS general symptoms
- Figure S7. Stability of correlation of epicenter values at the individual level to PANSS total score
- Figure S8. Stability of correlation of epicenter values at the individual level to chlorpromazine analogues
- Figure S9. Stability of correlation of epicenter values at the individual level to duration of illness
- Cross-modality generalizability of hub and epicenter models in surface area alterations of schizophrenia
  - Surface area mega-analysis
  - Functional and structural degree centrality predict regional susceptibility to surface area alterations
  - Disease epicenters of schizophrenia-related surface area alterations
  - Figure S10. Cross-modality generalizability of mega- and network modeling analysis in surface area
  - Table S19. Cortical surface area differences between schizophrenia patients and healthy controls
  - Table S20. Schizophrenia cortical surface area epicenters
  - Subject-level correlations individual hub vulnerability and epicenters of surface area alterations
  - Figure S11. Stability of correlation of hub vulnerability and clinical variables at the individual level
  - Table S21. Correlations between subject-level surface area functional and structural hub vulnerability and clinical scores
  - Table S22. Correlations between individual subject-level functional surface area epicenters and clinical scores
  - Table S23. Correlations between individual subject-level functional surface area epicenters and clinical scores
  - Figure S12. Cross-disorder comparison of surface area network modeling
- Acknowledgements per dataset
- References

Table S1. Site-specific inclusion criteria

Cohort	Country	Diagnosis measurement	Sample characteristics/inclusion criteria	Exclusion criteria
**ASRB**	Australia	Diagnosis was confirmed using the OPCRIT algorithm applied to interviewer ratings on the DIP, acc. to ICD-10 criteria	All participants were fluent English speakers and aged 18-65 years old	No history of an organic brain disorder, brain injury accompanied by > 24 h of amnesia, mental retardation defined as an IQ < 70, movement disorder, current substance dependence, or electro- convulsive therapy in the preceding 6 months. The control participants additionally had no personal history of psychotic disorder or family history of psychotic disorder in their first-degree biological relatives.
**CAMH**	Canada	SCID DSM-IV-TR Axis I	Schizophrenia outpatients who were clinically stable as determined by no medication change within the past month	Exclusion criteria were intelligence quotient < 70 as estimated by the Wechsler Test for Adult Reading (WTAR), substance dependence or abuse reported or indicated by a urine toxicology screen, head trauma with loss of consciousness, neurological disorders, and any magnetic resonance imaging contraindications. A first degree relative with a primary psychotic disorder was also an exclusion criterion for controls.
**CIAM**	South Africa	SCID DSM-IV-TR Axis I	Stable outpatients between ages 19-40 years with a diagnosis of schizophrenia or bipolar type I disorder with psychotic features or methamphetamine induced psychotic disorder or controls without a history or family history of psychotic symptoms.	Patients were excluded if they had a psychotic disorder other than schizophrenia or bipolar type I with psychotic features or methamphetamine induced psychotic disorder (i.e., schizophreniform disorder). Patients or controls were excluded if they had a physical condition requiring medication, prior head trauma or neurosurgery, any history of a cardiovascular event, a history or family history of epilepsy, a learning disability, if they were pregnant or lactating, or had any metal brain implants. Patients with bipolar type II disorder were excluded. Patients in the methamphetamine psychosis group were excluded if there was any evidence of symptoms persisting longer than 1 month after the cessation of methamphetamine use or if there was evidence of prior psychotic symptoms not related to the use of methamphetamine.
**COBRE**	USA	SCID DSM-IV Axis I Disorders	All participants were in the 18-65 age range and had a diagnosis of schizophrenia. Healthy individuals were included if they did not have a personal or family history of psychiatric disorders.	History of neurological disorder, history of mental retardation, history of severe head trauma with more than 5 minutes loss of consciousness, history of substance abuse or dependence within the last 12 months and MRI contraindications.
**ESO**	Czech Republic	ICD-10 (F20.x, F23, F25)	Early or first-episode psychosis, Czech language as a mother tongue, 18-60 years old	Neurocognitive disorders (organic mental disorder), mental disorders caused by addiction, mental retardation (IQ<80), severe neurological disorder, head injury, hypertension, cerebrovascular disease, epilepsy, migraine, endocrine disorders.
**FOR210 Marburg**	Germany	Semi-structured interview using SCID DSM-IV-TR Axis-I Disorders	All participants were aged between 18-65 and were fluent German speakers. Patients (in-and out-patients) had a	Exclusion criteria were any history of neurological (head trauma or unconsciousness) andmedical condition (severe somatic disorders), magnetic resonance imaging

			lifetime diagnosis of schizophrenia. Healthy individuals were included if they did not have any lifetime history of psychiatric disorders.	contraindications, verbal IQ < 80 (assessed using MWT-B), current substance dependence or benzodiazepine treatment.
**FOR210 Muenster**	Germany	Semi-structured interview using SCID DSM-IV-TR Axis-I Disorders	All participants were aged between 18- 65 and were fluent German speakers. Patients (in-and out-patients) had a lifetime diagnosis of schizophrenia. Healthy individuals were included if they did not have any lifetime history of psychiatric disorders.	Exclusion criteria were any history of neurological (head trauma or unconsciousness) and medical condition (severe somatic disorders), magnetic resonance imaging contraindications, verbal IQ < 80 (assessed using MWT-B), current substance dependence or benzodiazepine treatment.
**FIDMAG**	Spain	DSM-IV criteria based on interview and review of clinical history	Patients had a diagnosis of schizophrenia. All participants were in the 18-65 age range.	Controls were excluded if they reported a history of mental illness and/or treatment with psychotropic medication. Patients were excluded if have had a history of brain trauma or neurological disease or had shown alcohol/ substance abuse within 12 months before participation
**FSLRome**	Italy	SCID DSM-IV Axis I Disorders (SCID-I) andSCID DSM-IV Axis II Personality Disorders (SCID-II)	Inclusion criteria were (i) age between 18 and 65years; (ii) at least five years of education; and (iii) suitability for MRI scanning.	Exclusion criteria were (i) history of alcohol or drug abuse in the two years before the assessment; (ii) lifetime drug dependence; (iii) traumatic head injury with loss of consciousness; (iv) past or present major medical illness or neurological disorders; (v) any (for HC) or additional (for patients) psychiatric disorder or mental retardation; (vi) dementia or cognitive deterioration according to DSM-IV-TR criteria, and Mini-Mental State Examination (MMSE) score<25, consistent with normative data in the Italian population; (vii) not able and willing to give written informed consent.
**GIPSI**	Colombia	DSM-IV-TR diagnosis criteria using the Diagnostic Interview for Genetic Studies (DIGS)	Subjects with diagnosis of Schizophrenia, between the ages of18 and 60 years old.	History of traumatic brain injury, personality disorders or autism spectrum disorders.
**IGP**	Australia	Diagnosis was confirmed using the OPCRIT algorithm applied to interviewer ratings on the DIP, acc. to ICD-10 criteria	All participants were fluent English speakers and aged 18-65 years old	General exclusion criteria included an inability to communicate sufficiently in English, a current neurological disorder, a diagnosis of substance abuse or dependence in the pastsix months; and/or having been treated with electroconvulsive therapy in the previous six months.
**MCIC**	USA	SCID DSM-IV (SCID-NP for controls) or CASH were used to diagnose primary and co-morbid psychiatricdisorders in controls and patients	All subjects were between the ages of 18 and 60 and spoke English as their native language. To be included in the schizophrenia cohort, patients had to meet diagnostic criteria for schizophrenia, schizoaffective disorder, or schizophreniform disorder. Concerted effort was made to recruit	Control subjects who met criteria for current or past history of substance abuse or dependence were excluded from the study. Patients, however, were not excluded from the study unless criteria were met for current (i.e., within the past month) abuse or dependence (except for 6 patients who were found to meet criteria for current abuse after the study data was collected). Both patients and controls were excluded if they had (1) an IQ less than 70 based on a standardized IQ test, (2) history of a head injury resulting in prolonged loss of consciousness, neurosurgical procedure, neurological disease, history of skull fracture, severe or disabling medical conditions, or (3) a contraindication for MRI scanning such as

			patients early in the course of their illness and especially those who were antipsychotic drug naïve. The healthy control subjects with no current or past history of psychiatric illness including substance abuse or dependence were matched within site to the patient cohort for age, sex, and parental education. Control subjects who had not been diagnosed with any psychiatric disorders, but had been medicated with antidepressants, anti- anxiety medication or medication for sleep disturbance were included in the study provided that the duration of their medication did not exceed 2 months of lifetime use and no medication was used within the 6 months preceding the baseline MRI scan.	pregnancy, metal in body or head including implanted pacemaker, medication pump, vagal stimulator, deep brain stimulator, implanted TENS unit, or ventriculo -peritoneal shunt
**MPRC**	USA	SCID DSM-IV combined with a review of medical records	Individuals diagnosed with schizophrenia or schizoaffective disorder; and healthy controls without current DSM-IV Axis I psychiatric illnesses.	The exclusion criteria included diagnosis with hypertension, hyperlipidemia, type 2 diabetes, heart disorders, major neurologic event such as stroke or transient ischemic attack, and recent substance use disorder (except tobacco and marijuana use).
**OLIN**	USA	SCID DSM-IV	**AA:** Participants (ages 18-70) were healthy controls and individuals with a DSM-IV diagnosis of schizophrenia or schizoaffective disorder. Healthy controls were allowed to have common psychiatric disorders (exceptfor any type of psychosis).<br>**BSNIP:** Participants (ages 15-65) were recruited from 5 sites (Hartford, Baltimore, Chicago, Dallas, Boston) and included healthy controls, individuals with a DSM-IV diagnosisof schizophrenia, schizoaffective disorder. Healthy controls had no	**AA:** Exclusion criteria for all subjects included a history of major medical disorders, severe head injury, MRI contraindication, IQ < 70, dementia, traces of drugs (excluding THC) in urine, or drug intoxication during cognitive or MRI assessment.<br>**BSNIP:** Exclusion criteria for all subjects included history of seizures or head injury with loss of consciousness >10 minutes; positive urine drug screen for common drugs of abuse on the day of testing; diagnosis of substance abuse in the past 30 days or substance dependence in the past 6 months; history of systemic medical or neurological disorder likely to affect cognitive abilities; age-corrected Wide-Range Achievement Test, 4th edition, reading test standard score <65; and < 6th grade English reading level.<br>**BPP:** Exclusion criteria for all subjects included alcohol or drug abuse or dependence within the past 6 months, a history of major medical or neurological disorders, or IQ <70 as assessed by the WAIS.

			personal or family history (first degree) of psychotic disorders; no personal history of recurrent mood disorder; no lifetime history of substance dependence; and no history of any significant cluster A Axis II personality features defined bymeeting full criteria or within 1 criterion of a cluster A diagnosis usingthe Structured Interview for DSM-IV Personality. We provided healthy control and SZ data only from theHartford site.<br>**BPP:** Participants (ages 18-70) were healthy controls. Healthy control subjects were included if they had nolifetime history of axis I psychiatric disorders as assessed by the SCIDand no family history of mood or psychotic disorders.	
**PAFIP**	Spain	SCID DSM-IV for patients confirmed by an independent psychiatrist 6months after the initial contact. CASH for controls.	Patients had to meet the following criteria: (1) age 15–60 years; (2) living in the catchment area; (3) experiencing a first episode of psychosis; (4) no prior treatment with antipsychotic medication or, if previously treated, a total lifetime of adequate antipsychotic treatment of less than 6 weeks; and (5) meeting DSM-IV criteria for schizophrenia, schizophreniform disorder, briefpsychotic disorder, or schizoaffective disorder.	Patients were excluded when meeting DSM-IV criteria for (1) drug dependence (except nicotine dependence), (2) mental retardation, and when having a history of neurological disease or head injury. Controls exclusion criteria were current or past history of psychiatric, neurological or general medical illnesses, including substance dependence and significant loss of consciousness. HCs were selected to have a similar distribution in age, sex, laterality index, drug history and years of education as the patient population. The absence of psychosis in first-degree relatives was also confirmed by clinical records and family interview. After a detailed description of the study, each subject gave written informed consent to participate.
**PENS**	USA	SCID for DSM-IV and DIGS Psychosis module performed by a trained clinical interviewer. Then all clinical materials	The sample included individuals with a schizophrenia spectrum disorder (n = 35) or bipolar disorder, first degree biological relatives of persons with a schizophrenia spectrum or bipolar	Participants were native English speakers, 18 to 60 years old, with normal or corrected hearing and vision, and IQ of at least 70. Participantswith a history of intellectual disability were excluded. Patients and controls were additionally excluded for substance abuse or dependence. within the past 6 months; history of electroconvulsive therapy, epilepsy,

		reviewed by doctoral and graduate level psychologists to achieve a consensus diagnosis.	disorder, and healthy controls. Participants were recruited from the Minneapolis Veterans Affairs Health Care System (VAHCS) and mental health centers in the Minneapolis community as part of a larger research protocol that included neurocognitive, MRI, and additional electroencephalography procedures.	diagnosed seizure disorder, stroke, or neurological condition; uncontrolled medical condition likely to substantially affect brain functioning (e.g., untreated thyroid condition); and head injury resulting in fractured skull or more than 30 minutes unconsciousness. Healthy controlswere also excluded for history of primary psychotic disorder or hypomania, antipsychotic medication use, current or past depressive episodes, attention-deficit/hyperactivity disorder or other learning disability, and family history of bipolar or psychotic disorder.
**PHCP**	USA	SCID for DSM-IV and DIGS Psychosis module performed by a trained clinical interviewer. Then all clinical materials reviewed by doctoral and graduate level psychologists to achieve a consensus diagnosis.	People with Psychosis (PwP) were between the ages of 18 and 65 years old with a diagnosis of schizophrenia, schizoaffective disorder, or bipolar I disorder with a history of psychotic symptomatology (i.e., delusions or hallucinations) with no indication that symptoms were caused by substance use or a general medical condition. While PwP were screened and excluded for current substance use issues, a history of such issues as well as current/lifetime comorbidities of any kind were permitted for enrollment in the study in order to have a sample representative of patients with psychosis in the general population while simultaneously limiting nuisance. effects.	To be eligible for enrollment, all participants spoke English as their primary language and did not have: a legal guardian (or otherwise lack capacity to provide informed consent), alcohol/drug abuse in the past month or alcohol/drug dependence in the last 6 months, a diagnosed Learning Disability or estimated IQ lower than 70 (if either condition was diagnosed based on testing by a trained professional or the latter by research staff), a current or past central nervous system disease (including: seizures, epilepsy, encephalitis, MS, Parkinson's, stroke), his- tory of head injury with skull fracture or loss of consciousness greater than 30 min, history of electro-convulsive therapy (ECT) in the last year, tardive dyskinesia (as evidenced by medical record), obstructed or com- promised vision (e.g., lazy eye that is uncorrected or was corrected after age 17 / strabismus / cross eyes / permanent eye injury / abnormality in visual field / cataract), hearing problems (e.g., cannot hear with- out hearing aid / severe tinnitus), or a condition likely making it impossibleto perform tasks (e.g., paralysis, severe arthritis).
**RSCZ**	Russian Federation	ICD-10 (F20.x)	Early or first-episode psychosis in-patients (no later than 5 years since the first episode) who were clinically stable and received antipsychotic medication therapy. Mentally healthy controls were recruited from acquaintances of the researchers and clinical staff. All participants were fluent Russian speakers, right-handed males.	Common exclusion criteria for patients and controls were: organic brain disorders, neurological or severe somatic disorders, mental retardation, alcohol or substance abuse, history of head trauma with loss of consciousness for more than 5 min. In addition, controls were excluded if they had a family history of psychiatric illness.

**SCORE**	Switzerland	ICD-10 or DSM-IV criteria	First-episode psychosis patients who fulfilled criteria for brief psychotic disorder. All patients were between 18 and 42 years of age.	History of previous psychotic disorder, psychotic symptoms secondary to an organic disorder, substance abuse (except nicotine), psychotic symptoms associated with an affective psychosis or a borderline personality disorder, age younger than 18 years, inadequate knowledge of the German language, and IQ less than 70 as measured by the Mehrfachwahl Wortschatz Test Form B.
**Singapore**	Singapore	SCID DSM-IV	Inclusion criteria include: - 1) DSM IV diagnosis of SZ (Patients) 2) Age: 21- 65 3) English speaking 4) Provision ofinformed written consent	Exclusion criteria include: - 1) History of significant head injury 2) Significant Neurological diseases (such as epilepsy, cerebrovascular accident) or Medical Illnesses 4) Significant DSM IV alcohol or substance use or dependence 6) Contraindications to MRI (e.g., pacemaker, orbital foreign body, recent surgery/procedure with metallic devices/implants deployed) 7) Pregnant women 8) Claustrophobia
**SWIFT**	Switzerland	SCID DSM-IV-TR	Age between 18-65 y/o, good German language skills that allowed them to understand the consent procedure and to undergo the clinical assessment, right-handedness according to the Edinburgh Inventory. For patients: Diagnosis with a schizophrenia spectrum disorder.	Participants were excluded if they were left-handed, pregnant, showed any contraindications for MRI (e.g. metal-containing implants such as pacemaker or cochlear implants, claustrophobia), had a history of serious neurological issues, or reported current abuse of alcohol and/or psychoactive substances (apart from nicotine). Additionally, controls had no current major psychiatric DSM-IV Axis I diagnoses, as assessed with the screening questionnaire of the Structured Clinical Interview for DSM-IV Axis I Disorders.
**UCISZ**	USA	SCID DSM-IV-TR criteria	All subjects diagnosed with schizophrenia were clinically stable outpatients whose antipsychotic medications and doses had not changed within the last two months.	Schizophrenia and healthy volunteers with a history of major medical illness, drug dependence in the last five years (except for nicotine), current substance abuse disorder, or MRI contraindications, were excluded. Individuals with schizophrenia who had significant tardive dyskinesia and healthy volunteers with a current or past history of major neurological or psychiatric illness or with a first-degree relative with an Axis-I psychotic disorder diagnosis were also excluded.
**UPenn**	USA	SCID DSM-IV-TR criteria	A DSM diagnosis of schizophrenia or schizoaffective disorder	Participants were excluded if they had a history of major medical illness that could impact brain function, active substance misuse, or a contraindication to MRI.
**Zurich**	Switzerland	MINI DSM IV	A diagnosis of schizophrenia	We excluded patients with any other DSM-IV Axis I disorder (in particular, current substance use disorder and major depressive disorder), those medicated with lorazepamat a dose higher than 1 mg, those with florid psychotic symptoms (i.e., any positive subscale item scores higher than 4 on the PANSS scale and those with extrapyramidal side effects (i.e., a total score higher than 2 on the MSAS). Healthy controls were screened for any neuropsychiatric disorders using the structured Mini-International Neuropsychiatric Interview to ensure that they had no previous or present psychiatric illness. Both patients and healthy controls were required to have a normal physical and neurologic status and no history of major head injury or neurologic disorder

SCID: Structured Clinical Interview for DSM Disorders; CAMH: Comprehensive Assessment of Symptoms and History; MINI DSM IV: Mini-International Neuropsychiatric Interview for DSM IV; OPCRIT: Operational Criteria Checklist for Psychotic Illness and Affective Illness; DIP: Diagnostic Interview for Psychosis; ICD: international classification of disease; PANSS: Positive and Negative Syndrome Scale; MSAS: Modified Simpson–Angus Scale.

Table S2: Site demographics

Site	N total	N SCZ	N HC	Age SZ	Age HC	%M/%F SCZ	%M/% F HC	Mean Duration Illness SZ	PANSS Positive	PANSS Negative	SAPS Total	SANS Total
ASRB	429	263	166	38.6	39.3	67.3/32.7	47.6/52.4	15	NA	NA	NA	18.5
CAMH	264	118	146	43.9	43.6	59.3/40.7	52.7/47.3	19.2	13.9	14	NA	NA
CIAM	51	21	30	31	26.6	61.9/38.1	53.3/46.7	8.3	13.6	15.2	NA	NA
COBRE	143	73	70	37.4	35.7	82.2/17.8	71.4/28.6	15.8	15.2	14.8	NA	NA
ESO	80	40	40	29.4	29.1	50/50	50/50	0.6	14.2	16.1	NA	NA
fidmag	283	160	123	39.6	37.5	77.5/22.5	43.9/56.1	15.5	16.8	22.6	NA	37.2
FOR210Marburg	403	37	366	37.2	34	62.2/37.8	39.1/60.9	15.9	NA	NA	13.2	18.8
FOR210Muenster	163	8	155	33.4	27	50/50	38.7/61.3	11.1	NA	NA	6.4	8.1
FSL_Rome	280	164	116	39.4	37.5	67.1/32.9	62.9/37.1	14.9	20.9	21	31.5	28.8
GIPSI	43	43	0	33.5	NA	81.4/18.6	NA/NA	14.1	NA	NA	9.3	32.2
IGP	138	68	70	41.7	36	58.8/41.2	54.3/45.7	18.8	13.8	14.5	13	6.9
MCIC	213	117	96	33.9	32.7	74.4/25.6	67.7/32.3	11.1	NA	NA	NA	NA
MPRC	500	230	270	36.4	37.1	61.3/38.7	43.7/56.3	NA	NA	NA	NA	NA
OLIN	523	135	388	36.6	37.8	66.7/33.3	47.7/52.3	14.5	7.7	7.9	NA	NA
PAFIP1.5T	222	142	80	29.7	27.7	62/38	62.5/37.5	1	NA	NA	13.6	6.4

PAFIP3T	217	114	103	29.7	30.1	56.1/43.9	60.2/39.8	0.7	NA	NA	14.1	5.5
PENS	51	17	34	48.4	46.8	70.6/29.4	44.1/55.9	24.7	NA	NA	12.2	17.8
PHCP	129	47	82	43.9	43.8	70.2/29.8	43.9/56.1	18.4	NA	NA	13.9	23.4
RSCZ_data	98	46	52	22.2	22.3	100/0	100/0	1.1	11.2	18.5	NA	NA
SCORE	127	72	55	26.9	26	72.2/27.8	45.5/54.5	NA	NA	NA	NA	8.15
Singapore	227	151	76	33.1	31.8	69.5/30.5	61.8/38.2	6.5	10.6	9	NA	NA
STGO	170	85	85	19.8	23.1	82.4/17.6	68.2/31.8	0.1	16.1	21.3	NA	NA
SWIFT	37	24	13	34.2	29.3	70.8/29.2	38.5/61.5	9.5	16.4	12.8	NA	NA
UCISZ	57	27	30	42.9	41.4	81.5/18.5	76.7/23.3	17.5	15.6	16	13.4	22.8
UPenn	370	177	193	38.9	36.4	59.3/40.7	46.6/53.4	17.3	NA	NA	18.3	23.7
Zurich	88	60	28	30.5	32.5	75/25	64.3/35.7	8.4	10.7	14.5	NA	24.9

Table S3. Sample image acquisition and image pre-processing details by cohort

Cohort	Number of scanners	Scanner Vendor & Type	Imaging Protocols	Slice orientation	FreeSurfer Version	Operating System	Number of subjects removed from analysis due to QC failure with reasons
ASRB	5	Siemens Avanto 1.5T	High-resolution T1-weighted structural magnetic resonance imaging (sMRI) brain scans (MPRAGE) were acquired using an optimized magnetizationprepared rapid acquisition gradient echo on 1.5 T Siemens Avanto scanners (Siemens, Erlangen, Germany) across five Australian research sites (Loughland and al., 2010). Image parameters were set to 176 slices of 1mm thickness, no gap with field-ofview 250 x 250 mm2, repetition time 1980 ms, echo time 4.3 ms, data acquisition matrix 256 x 256, with a flip matrix of 15°, resulting in a voxel size of 0.98×0.98×1.0 mm3	Sagittal	v5.1.0	Mac OSX	0
CAMH	1	GE 1.5T	SPGR, TR/TE/TI=12.3/5.3/300ms, flip angle=20°, 256x256x128 matrix, FOV=240x240mm, slice thickness=1.5mm	Axial	v5.3.0	xubuntu x86_64-linux	0
CIAM	7	3T Siemens Allegra	Sequence: 3D T1-weighted magnetization prepared rapid, Direction: Sagittal, Slices: 129, Gap: (mm) 0, Voxels: (mm) 1.3 x 1.0 x 1.3, TE: (ms) 1.53; 3.21; 4.89; 6.57, TR: (ms) 2530, Flip: angle: 7	Sagittal	v.5.3.0	NA	0
COBRE	1	3T Siemens TIM Trio	T1-weighted images were acquired with a 5-echo multi-echo MPRAGE sequence [TE (echo times) = 1.64, 3.5, 5.36, 7.22, 9.08 ms, TR (repetition time) = 2.53 s, TI (inversion time) = 1.2 s, 7○ flip angle, number of excitations (NEX) = 1, slice thickness = 1 mm, FOV (field of view) = 256 mm, resolution = 256x256]	Sagittal	v5.3.0	Linux RedHat	A total of 9 participants were excluded following ENIGMA QA protocol.<br><br>9 total: 6 SCZ, 3 HC; 8 Males; average age: 37.89 (SD = 10.30). age range: 25-52 y.o.

ESO	1	3T Siemens Tim Trio	MP-RAGE 3D, 1mm thickness, acquisition matrix 256 x 256, TR=2300ms, TE=4.63ms,TI=900ms	Sagittal	v5.3.0	Linux	Only subjects without significant motion artifacts (assessed by visual inspection) were included. Apart from ENIGMA QA protocol, visual inspection of all slices and edits to the skullstrip, white matter segmentation and control points insertion for correction of signal intensity normalization were done where needed. No subjects were excluded.
FIDMAG	1	1.5T GE Signa	180 axial slices; 1mm slice thickness, no gap, matrix size 512x512; 0.5x0.5x1mm3 voxel resolution; TE 4ms, TR 2000ms, flip angle 15	Axial	v5.3.0	Linux Ubuntu	0
FOR210 Marburg		3T Siemens Magnetom Trio	Flip angle 9, TR (ms) 1900, TE (ms) 2.26, TI (ms) 900, Voxels (mm) 1 x 1 x 1, Gap (mm) 0.5, Slices 176, Direction, 3D T1-weighted magnetization	Sagittal	v5.3.0		
FOR210 Muenster		3T Siemens PRISMA	Flip angle 8, TR (ms) 2130, TE (ms) 2.28, TI (ms) 900, Voxels (mm) 1 x 1 x 1, Gap (mm) 0, Slices 192, 3D T1-weighted magnetization prepared rapid	Sagittal	v5.3.0		
FSLRome	1	Siemens 3T Allegra	3D MPRAGE: TE/TR=2.4/7.92 ms, flip angle=15°, voxel size 1×1×1 mm	Sagittal	6.0dev	Linux	0
GIPSI	1	3T Philips Achieva Philips	Flip angle 8, TR (ms) 4.76, TE (ms) 2.06, TI (ms) NA, Voxels (mm) 1 x .6 x .6, Gap (mm) 0, Slices 160, , Sequence 3D T1-weighted TFE	Axial	v5.0.0		
IGP	1	Philips 3T Achieva TX	TR 8.9ms, TE 4.1ms, field of view 240mm, matrix 268 x 268, 200 sagittal slices, slice thickness 0.9mm, no gap	Sagittal	v5.3.0	Mac OSX	0
MCIC	3	1.5, 3T Siemens and GE	T1 scans: TR = 2530 ms for 3 T, TR = 12 ms for 1.5 T; TE = 3.79 ms for 3 T, TE = 4.76 ms for 1.5 T; FA = 7 for 3 T, FA = 20 for 1.5 T; TI = 1100 for 3 T; Bandwidth = 181 for 3 T, Bandwidth = 110 for 1.5 T; 0.625×0.625 mm voxel size; slice thickness 1.5 mm; FOV 256×256×128 cm matrix; FOV = 16 cm (could be increased to 18 cm for full brain coverage).	Coronal	v4.0.1	Linux of various flavors	5 subjects failed automated segmentation procedure due to excessive motion artifacts 2 participants' MRI data failed the manual inspection

MPRC	2	MPRC 1:<br>3T Siemens Allegro<br><br>MPRC 2:<br>3T Siemens Trio	MPRC 1: T1-weighted, 3D MPRAGE, 1x1x1mm, TE/TR/TI=4.3/2500/1000ms, flip angle=8 degrees.<br><br>MPRC 2**:** T1-weighted, 3D MPRAGE, 1x1x1mm, TE/TR/TI=2.9/2300/900ms, flip angle=9 degrees.	Sagittal	v5.3.0	Linux	0
OLIN	1	3T Alegra	1.25 mm, 5:36 min scan time each<br><br>T1-weighted, 3D magnetization prepared rapid gradient-echo (MPRAGE) sequence (TR/TE/TI=2200/4.13/766 ms, flip angle=13°, voxel size [isotropic]=0.8mm, image size=240 320 x 208 voxels), with axial slices parallel to the AC-PC line.	Axial	v5.1.0		
PAFIP	2	GE 1.5T	Three-dimensional T1-weighted images, using a spoiled grass (SPGR) sequence acquired in the coronal plane with: echo time (TE)=5 ms, repetition time (TR)=24 ms, numbers of excitations (NEX)=2, rotation angle=45°, field of view (FOV)=26×19.5 cm, slice thickness=1.5mm and a matrix of 256×192.	Coronal	v5.0.0	Ubuntu<br>11,04 (x86_64)	1 subject was excluded because motion artifacts resulted in very poor segmentation
PENS	1	Siemens 3 T Prisma scanner with a Siemens 32 channel head coil	Structural MRI using a 10-min T1-weighted MPRAGE sequence (TE = 2.12 ms, TR = 2,400 ms, flip angle = 8, resolution = 256)	Sagittal	V6.0		
PHCP	1	Siemens 3 T Prisma scanner with a Siemens 32 channel head coil	A multi-echo T1w MPRAGE sequence and a variable-flip-angle, turbo-spinecho T2w scan with volumetric navigators to aid real-time motion correction and selective reacquisition were acquired with scanning protocol identical to that of the Lifespan Human Connectome up to 30 k-space lines for the<br>T1w scan and up to 25 k -space lines for the T2w scan were allotted for reacquisition. T1w MPRAGE multi-echo (300 × 320 matrix, FOV=240 × 256mm, resolution=0.8mm, flip angle=8, TE=1.81, 3.6, 5.39, 7.18 ms, TR=2500ms, slices/orientation=208 sag,	Sagittal	V6.0		

			AF=2, time=8min:22sec				
RSCZ	1	3T Philips Achieva	A turbo field echo sequence covering the whole brain. TR = 8.2 ms, TE = 3.7 ms, flip angle = 8, FOV = 240 mm, voxel size of 0.83 × 0.83 mm with a slice thickness of 1 mm, no gap.	Sagittal	v5.3.0	Centos 6.6	0. Only subjects without significant motion and other artifacts (assessed by visual inspection) were included for carrying out ENIGMA QA protocol.
SCORE	1	3T Philips Achieva	MPRAGE: aquicition matrix: 256×256×176, isotropic spatial resolution: 1x1x1mm3, TI=1000ms, TR=2s, TE=3.4 ms, flip angle: 8° and bandwidth of 200 Hz/pixel	Sagittal	6.0dev	ubuntu 18.04 LTS	From the initial data set, 11 subjects had to be excluded based on erroneous brain segmentation. Among them, 4 subjects revealed statistical outliers.
IMH Singapore	1	3T Philips Achieva	T1 scans: 180 axial slices of 0.9mm thickness with no gap, FOV = 230x230 mm2, matrix 256x204, voxel size =0.89x0.89x0.9 mm3, TR=7.2 s, TE=3.3 ms, FA=8°,	Axial	v5.3.0	Mac OSX	2 due to excessive motion artifacts, SZ=1 (male, age 33 years), HC=1 (male, 31 years)
SCORE	1	3T Siemens	MPRAGE: aquicition matrix: 256×256×176, isotropic spatial resolution: 1x1x1mm3, TI=1000ms, TR=2s, TE=3.4 ms, flip angle: 8° and bandwidth of 200 Hz/pixel	Sagittal	6.0dev		
SWIFT	1	3T Siemens Verio	MPRAGE: 160 sagittal slices, 1mm slice thickness, 256x256 matrix size, 1x1x1 mm3 voxel size. TR = 2.3ms, TE = 2.98ms, TI = 900ms.	Sagittal	v 7.1.1	Linux	0
UCISZ	1	3T Philips Achieva	T1TFE:200 sagittal slices, 320x274 matrix size, .75mm isotropic, TR = 11ms, TE=4.562ms, flip angle = 18°,	Sagittal	v6.0dev	Centos 3.10.72-1.el6.elrepo.x86_64	0
UNIBA	1	3T GE	T1-weighted 3D FFE, TR/TE 9.86/4.6ms, 0.875x0.875x1 voxels, flip angle 8, FOV 224x160x168, 160 slices	Axial	v5.3.0	Ubuntu 10.04, Kernel Linux 2.6.32-25-generic, GNOME 2.30.2	3 due to motion artefacts
Zurich	1	3T Philips	3D T1-weighted images were acquired with an ultra fast gradient echo T1-weighted sequence(TR=8.4ms, TE=3.8ms, flip angle=8°) in 160 sagittal plan slices (1mm slice thickness, no slice gap) of 240×240mm2 resulting in 1x1x1mm3voxels.	Sagittal	v6.0.0	Linux	0

HCP MRI data and preprocessing

HCP data were acquired on a Siemens Skyra 3T and included (i) T1-weighted images [magnetization-prepared rapid gradient echo sequence, repetition time (TR) = 2400 ms, echo time (TE) = 2.14 ms, field of view (FOV) = 224 × 224 mm2, voxel size = 0.7 mm3, 256 slices], (ii) resting-state fMRI [gradient-echo echo-planar imaging (EPI) sequence, TR = 720 ms, TE = 33.1 ms, FOV = 208 × 180 mm^2, voxel size = 2 mm3, 72 slices], and (iii) diffusion MRI (spin-echo EPI sequence, TR = 5520 ms, TE = 89.5 ms, FOV = 210 × 180mm^2, voxel size = 1.25 mm3, b-value = 1000/2000/3000 s/mm2, 270 diffusion directions, 18 b0 images). HCP data underwent the initiative's minimal preprocessing (1, 2). Resting-state fMRI data underwent distortion and head motion corrections, magnetic field bias correction, skull removal, intensity normalization, and were mapped to MNI152 space. Noise components attributed to head movement, white matter, cardiac pulsation, arterial, and large vein–related contributions were automatically removed using ICA-FIX (3). Preprocessed time series were mapped to standard gray ordinate space using a cortical ribbon-constrained volume-to-surface mapping algorithm and subsequently concatenated to form a single time series. Diffusion MRI data underwent b0 intensity normalization and correction for susceptibility distortion, eddy currents, and head motion. High-resolution functional and structural data were parcellated according to the Desikan-Killiany atlas to align with the ENIGMA-Schizophrenia dataset (3).

Functional and structural connectivity matrix generation from HCP participants

Functional connectivity matrices were generated by computing pairwise correlations between the time series of all 68 cortical regions and between all subcortical and cortical regions; negative connections were set to zero. Subject-specific connectivity matrices were then *z*-transformed and aggregated across participants to construct a group-average functional connectome. To generate structural connectivity matrices, constrained tractography was performed using different tissue types derived from the T1w image, including cortical and subcortical gray matter, white matter, and cerebrospinal fluid (4). Multishell and multi-tissue response functions were estimated (5) and constrained spherical deconvolution and intensity normalization were performed (6, 7). The initial tractogram was generated with 40 million streamlines, with a maximum tract length of 250 and a fractional anisotropy cutoff of 0.06. Spherical-deconvolution informed filtering of tractograms (SIFT2) was applied to reconstruct whole-brain streamlines weighted by the cross-sectional multipliers (6). To produce normative subject-specific connectivity matrices according to the Desikan-Killiany atlas, we mapped reconstructed streamlines onto the 68 cortical and 14 subcortical (including hippocampus) regions (8). Normative structural connectivity matrices were generated from preprocessed diffusion MRI data using MRtrix3 (9). The group-average normative structural connectome was defined using a distance-dependent thresholding, which preserved the edge length

distribution in individual participants (10), and was log transformed to reduce connectivity strength variance. Hence, structural connectivity was defined by the number of streamlines between two regions (*i.e.,* fiber density). Our network centrality findings of healthy individuals reflect previously published results (11, 12) with centrality peaking in medial prefrontal, superior parietal and angular regions.

ComBat batch effect adjustment

In our original mega-analytic model, regional cortical thickness and subcortical volumes were the dependent variables whilst group (SCZ, HC), age and sex were the independent variables. To show that our batch-effect correction with ComBat on the structural MRI data was successful, we performed our mega-analytical linear regression with the ComBat adjusted and unadjusted data similar with the original model, hereby adding site as an independent variable. For each region, we then computed the variance explained by site as independent variable quantified by partial R^2. Partial R^2 was computed using the *rsq.partial* function of the *rsq* library in R. It can be seen on Table S4 that data adjustment with ComBat eliminates any variance explained by site as an independent variable, i.e., variation of imaging data due to site differences.

Table S4 ComBat batch effect adjustment confirmation

**Brain region**	**partial R^2 of site on ComBat-adjusted data**		**partial R^2 of site on ComBat unadjusted data**	
	**Left**	**Right**	**Left**	**Right**
bankssts_thickavg	0	0	0.29	0.30
caudalanteriorcingulate_thick	0	0	0.25	0.26
caudalmiddlefrontal_thickavg	0	0	0.31	0.30
cuneus_thickavg	0	0	0.28	0.26
entorhinal_thickavg	0	0	0.21	0.20
fusiform_thickavg	0	0	0.42	0.42
inferiorparietal_thickavg	0	0	0.29	0.36
inferiortemporal_thickavg	0	0	0.38	0.42
isthmuscingulate_thickavg	0	0	0.21	0.15
lateraloccipital_thickavg	0	0.001	0.32	0.35
lateralorbitofrontal_thickavg	0	0	0.24	0.24
lingual_thickavg	0	0	0.28	0.21
medialorbitofrontal_thickavg	0	0	0.30	0.29
middletemporal_thickavg	0	0	0.31	0.37
parahippocampal_thickavg	0	0	0.18	0.24
paracentral_thickavg	0	0	0.18	0.20
parsopercularis_thickavg	0	0	0.26	0.27
parsorbitalis_thickavg	0	0	0.13	0.16
parstriangularis_thickavg	0	0	0.21	0.26
pericalcarine_thickavg	0	0	0.42	0.36
postcentral_thickavg	0	0	0.30	0.30
posteriorcingulate_thickavg	0	0	0.23	0.21
precentral_thickavg	0	0	0.29	0.23
precuneus_thickavg	0	0	0.27	0.25
rostralanteriorcingulate_thic	0	0	0.28	0.29

rostralmiddlefrontal_thickavg	0	0	0.34	0.39
superiorfrontal_thickavg	0	0	0.31	0.28
superiorparietal_thickavg	0	0	0.34	0.35
superiortemporal_thickavg	0	0	0.34	0.36
supramarginal_thickavg	0	0	0.28	0.33
frontalpole_thickavg	0	0	0.17	0.17
temporalpole_thickavg	0	0	0.20	0.26
transversetemporal_thickavg	0	0	0.15	0.17
insula_thickavg	0	0	0.34	0.35

Table S5. Cortical thickness differences between schizophrenia patients and healthy controls

Regions	T-values	p values (Bonferroni-corrected)	Cohen's D	Cohen's D 95%CI
Left banks of superior temporal sulcus	12.26	1.44×10^{-34}	0.34	[0.28, 0.39]
Left caudal anterior cingulate cortex	3.01	8.95×10^{-4}	0.08	[0.03, 0.14]
Left caudal middle frontal gyrus	13.06	7.69×10^{-39}	0.36	[0.30, 0.41]
Left cuneus	6.71	7.21×10^{-12}	0.18	[0.13, 0.24]
Left entorhinal cortex	5.22	6.38×10^{-8}	0.14	[0.09, 0.20]
Left fusiform gyrus	14.46	5.42×10^{-47}	0.40	[0.34, 0.45]
Left inferior parietal cortex	14.38	1.64×10^{-46}	0.40	[0.34, 0.45]
Left inferior temporal gyrus	14.29	5.61×10^{-46}	0.39	[0.34, 0.45]
Left isthmus cingulate cortex	8.29	4.86×10^{-17}	0.23	[0.17, 0.28]
Left lateral occipital cortex	11.7	1.00×10^{-31}	0.32	[0.27, 0.38]
Left lateral orbitofrontal cortex	12.2	2.99×10^{-34}	0.34	[0.28, 0.39]
Left lingual gyrus	9.72	1.29×10^{-22}	0.27	[0.21, 0.32]
Left medial orbitofrontal cortex	6.86	2.64×10^{-12}	0.19	[0.13, 0.24]
Left middle temporal gyrus	15.26	5.52×10^{-52}	0.42	[0.36, 0.47]
Left parahippocampal gyrus	5.88	1.52×10^{-9}	0.16	[0.11, 0.22]
Left paracentral lobule	9.97	1.09×10^{-23}	0.27	[0.22, 0.33]
Left pars opercularis of inferior frontal gyrus	13.13	3.04×10^{-39}	0.36	[0.31, 0.41]
Left pars orbitalis of inferior frontal gyrus	10.85	1.26×10^{-27}	0.30	[0.24, 0.35]
Left pars triangularis of inferior frontal gyrus	11.81	3.01×10^{-32}	0.32	[0.27, 0.38]
Left pericalcarine cortex	2.34	6.55×10^{-3}	0.06	[0.01, 0.12]
Left postcentral gyrus	12.63	1.63×10^{-36}	0.35	[0.29, 0.40]
Left posterior cingulate cortex	10.02	6.90×10^{-24}	0.28	[0.22, 0.33]
Left precentral gyrus	12.71	5.88×10^{-37}	0.35	[0.29, 0.40]
Left precuneus	11.57	4.82×10^{-31}	0.32	[0.26, 0.37]
Left rostral anterior cingulate cortex	4.51	2.27×10^{-6}	0.12	[0.07, 0.18]
Left rostral middle frontal gyrus	11.95	5.59×10^{-33}	0.33	[0.27, 0.38]
Left superior frontal gyrus	13.83	3.22×10^{-43}	0.38	[0.33, 0.43]
Left superior parietal cortex	9.87	3.02×10^{-23}	0.27	[0.22, 0.33]
Left superior temporal gyrus	14.57	1.20×10^{-47}	0.40	[0.35, 0.45]
Left supramarginal gyrus	15.74	4.77×10^{-55}	0.43	[0.38, 0.49]
Left frontal pole	6.76	5.16×10^{-12}	0.19	[0.13, 0.24]
Left temporal pole	7.29	1.18×10^{-13}	0.20	[0.15, 0.25]

Left transverse temporal gyrus	9.16	$2.50x10^{-20}$	0.25	[0.20, 0.31]
Left insula	13.55	$1.32x10^{-41}$	0.37	[0.32, 0.43]
Right banks of superior temporal sulcus	12.12	$7.72x10^{-34}$	0.33	[0.28, 0.39]
Right caudal anterior cingulate cortex	5.05	$1.56x10^{-7}$	0.14	[0.08, 0.19]
Right caudal middle frontal gyrus	11.91	$8.93x10^{-33}$	0.33	[0.27, 0.38]
Right cuneus	7.64	$8.96x10^{-15}$	0.21	[0.16, 0.26]
Right entorhinal cortex	4.04	$1.84x10^{-5}$	0.11	[0.06, 0.16]
Right fusiform gyrus	15.12	$4.64x10^{-51}$	0.42	[0.36, 0.47]
Right inferior parietal cortex	13.03	$1.02x10^{-38}$	0.36	[0.30, 0.41]
Right inferior temporal gyrus	13.75	$9.58x10^{-43}$	0.38	[0.32, 0.43]
Right isthmus cingulate cortex	7.94	$8.22x10^{-16}$	0.22	[0.16, 0.27]
Right lateral occipital cortex	11.39	$3.71x10^{-30}$	0.31	[0.26, 0.37]
Right lateral orbitofrontal cortex	10.72	$5.36x10^{-27}$	0.29	[0.24, 0.35]
Right lingual gyrus	10.67	$8.45x10^{-27}$	0.29	[0.24, 0.35]
Right medial orbitofrontal cortex	7.45	$3.69x10^{-14}$	0.20	[0.15, 0.26]
Right middle temporal gyrus	13.9	$1.16x10^{-43}$	0.38	[0.33, 0.44]
Right parahippocampal gyrus	6.01	$6.70x10^{-10}$	0.17	[0.11, 0.22]
Right paracentral lobule	9.08	$5.31x10^{-20}$	0.25	[0.20, 0.30]
Right pars opercularis of inferior frontal gyrus	14.6	$8.08x10^{-48}$	0.40	[0.35, 0.46]
Right pars orbitalis of inferior frontal gyrus	10.31	$3.72x10^{-25}$	0.28	[0.23, 0.34]
Right pars triangularis of inferior frontal gyrus	12.16	$4.97x10^{-34}$	0.33	[0.28, 0.39]
Right pericalcarine cortex	2.32	$6.87x10^{-3}$	0.06	[0.01, 0.12]
Right postcentral gyrus	11.13	$6.59x10^{-29}$	0.31	[0.25, 0.36]
Right posterior cingulate cortex	9.34	$4.50x10^{-21}$	0.26	[0.20, 0.31]
Right precentral gyrus	11.72	$8.23x10^{-32}$	0.32	[0.27, 0.38]
Right precuneus	10.6	$1.91x10^{-26}$	0.29	[0.24, 0.35]
Right rostral anterior cingulate cortex	4.42	$3.46x10^{-6}$	0.12	[0.07, 0.18]
Right rostral middle frontal gyrus	11.53	$7.03x10^{-31}$	0.32	[0.26, 0.37]
Right superior frontal gyrus	12.82	$1.57x10^{-37}$	0.35	[0.30, 0.41]
Right superior parietal cortex	10.03	$6.31x10^{-24}$	0.28	[0.22, 0.33]
Right superior temporal gyrus	15.05	$1.30x10^{-50}$	0.41	[0.36, 0.47]
Right supramarginal gyrus	14.29	$6.06x10^{-46}$	0.39	[0.34, 0.45]
Right frontal pole	6.55	$2.18x10^{-11}$	0.18	[0.13, 0.23]
Right temporal pole	7.19	$2.45x10^{-13}$	0.20	[0.14, 0.25]
Right transverse temporal gyrus	9.4	$2.77x10^{-21}$	0.26	[0.20, 0.31]
Right insula	12.77	$2.95x10^{-37}$	0.35	[0.30, 0.40]

Table S6. Subcortical volume differences between schizophrenia and healthy controls

Regions	T-values	p values (Bonferroni-corrected)	Cohen's D	Cohen's D 95%CI
Laccumb	1.81	1	0.05	[0,0.1]
Lamyg	6.6	$4.55x10^{-11}$	0.18	[0.13,0.24]
Lcaud	-1.12	1	-0.03	[-0.08,0.02]
Lhippo	12.32	$2.05x10^{-34}$	0.34	[0.28,0.39]
Lpal	-9.56	1	-0.26	[-0.32,-0.21]

Lput	-4.41	1	-0.12	[-0.18,-0.07]
Lthal	9.74	$2.94x10^{-22}$	0.27	[0.21,0.32]
Raccumb	3.62	$2.95x10^{-4}$	0.1	[0.05,0.15]
Ramyg	6.58	$5.10x10^{-11}$	0.18	[0.13,0.23]
Rcaud	-1.64	1	-0.04	[-0.1,0.01]
Rhippo	12.52	$1.75x10^{-35}$	0.34	[0.29,0.4]
Rpal	-6.84	1	-0.19	[-0.24,-0.13]
Rput	-4.4	1	-0.12	[-0.17,-0.07]
Rthal	9.91	$5.60x10^{-23}$	0.27	[0.22,0.33]

Table S7. Schizophrenia cortical epicenter ranking (ordered by significance of functional epicenters)

Regions	Functional Epicenter R values	Functional Epicenter p_{spin} values (Bonferroni-corrected)	Structural Epicenter R values	Structural Epicenter p_{spin} values (Bonferroni - corrected)
Left entorhinal cortex	0.69	<.001	-0.08	1
Left banks of superior temporal sulcus	0.68	<.001	0.38	0.163
Left inferior temporal gyrus	0.67	<.001	0.24	1
Right inferior temporal gyrus	0.66	<.001	0.19	1
Right entorhinal cortex	0.65	<.001	0.01	1
Right banks of superior temporal sulcus	0.64	<.001	0.29	1
Left pars triangularis of inferior frontal gyrus	0.63	<.001	0.35	0.197
Left pars opercularis of inferior frontal gyrus	0.6	<.001	0.45	0.007
Right pars triangularis of inferior frontal gyrus	0.58	<.001	0.37	0.15
Right lateral orbitofrontal cortex	0.57	<.001	-0.11	1
Left caudal middle frontal gyrus	0.56	<.001	0.39	0.122
Left lateral orbitofrontal cortex	0.55	<.001	-0.07	1
Right pars orbitalis of inferior frontal gyrus	0.54	<.001	0.23	1
Left rostral middle frontal gyrus	0.54	<.001	0.22	1
Left supramarginal gyrus	0.54	<.001	0.36	0.435
Left pars orbitalis of inferior frontal gyrus	0.54	<.001	0.21	1
Right caudal middle frontal gyrus	0.51	<.001	0.33	0.653
Left superior temporal gyrus	0.51	<.001	0.22	1
Right superior frontal gyrus	0.5	<.001	-0.12	1
Right middle temporal gyrus	0.5	<.001	0.15	1
Left superior frontal gyrus	0.49	<.001	-0.09	1
Left middle temporal gyrus	0.48	<.001	0.32	0.313
Right superior temporal gyrus	0.47	<.001	0.26	1
Right pars opercularis of inferior frontal gyrus	0.46	.001	0.22	1
Right inferior parietal cortex	0.45	.001	0.34	0.333
Right rostral middle frontal gyrus	0.44	.001	0.19	1
Left inferior parietal cortex	0.44	.001	0.39	0.143
Left temporal pole	0.44	.002	0.21	1
Left transverse temporal gyrus	0.43	.002	0.21	1
Right supramarginal gyrus	0.43	.004	0.28	1

Right transverse temporal gyrus	0.41	.007	0.21	1
Left precentral gyrus	0.39	.008	0.37	0.299
Left posterior cingulate cortex	0.38	.009	-0.19	1
Left caudal anterior cingulate cortex	0.38	.009	-0.13	1
Left insula	0.37	.01	0.05	1
Right precentral gyrus	0.37	.012	0.31	1
Left fusiform gyrus	0.37	.012	-0.01	1
Right insula	0.36	.014	0.01	1
Left paracentral lobule	0.36	.017	0.06	1
Right paracentral lobule	0.35	.017	0.11	1
Left superior parietal cortex	0.35	.017	0.2	1
Right postcentral gyrus	0.34	.017	0.25	1
Right caudal anterior cingulate cortex	0.34	.019	-0.14	1
Right posterior cingulate cortex	0.34	.019	-0.14	1
Right temporal pole	0.33	.02	0.08	1
Right fusiform gyrus	0.32	.021	0.01	1
Right superior parietal cortex	0.32	.03	0.25	1
Left postcentral gyrus	0.31	.033	0.28	1
Left parahippocampal gyrus	0.29	.071	-0.08	1
Right parahippocampal gyrus	0.29	.075	-0.11	1
Right lateral occipital cortex	0.24	.081	0.07	1
Left lateral occipital cortex	0.24	.084	0.03	1
Right frontal pole	0.24	.084	0	1
Right precuneus	0.21	.142	-0.21	1
Left frontal pole	0.21	.182	-0.06	1
Left precuneus	0.18	.242	-0.24	1
Left isthmus cingulate cortex	0.13	.394	-0.27	1
Right isthmus cingulate cortex	0.11	.48	-0.26	1
Left pericalcarine cortex	0.11	.48	-0.07	1
Right pericalcarine cortex	0.1	.538	-0.14	1
Left cuneus	0.08	.637	-0.18	1
Left medial orbitofrontal cortex	0.07	.651	-0.09	1
Left lingual gyrus	0.06	.749	-0.08	1
Right cuneus	0.05	.766	-0.16	1
Right lingual gyrus	0.04	.773	-0.2	1
Left rostral anterior cingulate cortex	0.02	.8	-0.2	1
Right medial orbitofrontal cortex	-0.01	.903	-0.17	1
Right rostral anterior cingulate cortex	-0.05	.958	-0.16	1

Table S8. Schizophrenia subcortical epicenter ranking

Regions	Functional Epicenter R values	Functional Epicenter p_{spin} values (Bonferroni-corrected)	Structural Epicenter R values	Structural Epicenter p_{spin} values (Bonferroni - corrected)
L_Accumbens	-0.07	1	-0.16	1
L_Amygdala	0.53	<0.001	0.03	1
L_Caudate	0.47	0.001	0.16	1
L_Hippocampus	0.34	0.235	-0.05	1
L_Pallidum	0.42	0.014	0.21	1
L_Putamen	0.5	0.000	0.14	1
L_Thalamus	0.38	0.088	-0.02	1
R_Accumbens	-0.16	3.048	-0.23	1
R_Amygdala	0.48	0.001	-0.01	1
R_Caudate	0.43	0.010	-0.01	1
R_Hippocampus	0.32	0.428	-0.08	1
R_Pallidum	0.33	0.237	0.03	1
R_Putamen	0.46	0.001	0.13	1
R_Thalamus	0.33	0.309	0.18	1

Table S9. Divergent and convergent regions of different stages of SCZ (Note, no unique epicenters were found for chronic SCZ)

Divergent Regions		Convergent Regions
**First Episode Psychosis (FEP)**	**Early SCZ**	**FEP+Early SCZ + Chronic SCZ**
Functional Connectivity	*Functional Connectivity*	*Functional Connectivity*
Left cuneus	Left banks of superior temporal sulcus	Left inferior parietal cortex
Left fusiform gyrus	Left caudal middle frontal gyrus	Left inferior temporal gyrus
Left lateral occipital cortex	Left entorhinal cortex	Left lateral orbitofrontal cortex
Left lingual gyrus	Left middle temporal gyrus	Left pars opercularis of inferior frontal gyrus
Left pericalcarine cortex	Left pars orbitalis of inferior frontal gyrus	Left pars triangularis of inferior frontal gyrus
Left superior parietal cortex	Left posterior cingulate cortex	Left rostral middle frontal gyrus
Left superior temporal gyrus	Left superior frontal gyrus	Left supramarginal gyrus
Right caudal anterior cingulate cortex	Left insula	Right banks of superior temporal sulcus
Right lateral occipital cortex	Right caudal middle frontal gyrus	Right lateral orbitofrontal cortex
Right lingual gyrus	Right entorhinal cortex	Right pars triangularis of inferior frontal gyrus
Right pericalcarine cortex	Right inferior parietal cortex	
Right superior parietal cortex	Right inferior temporal gyrus	
Right transverse temporal gyrus	Right middle temporal gyrus	*Structural Connectivity*
	Right pars opercularis of inferior frontal gyrus	Left caudal middle frontal gyrus
Structural Connectivity	Right pars orbitalis of inferior frontal gyrus	Left pars opercularis of inferior frontal gyrus
Left banks of superior temporal sulcus	Right rostral middle frontal gyrus	Left precentral gyrus
Left cuneus	Right superior frontal gyrus	Right caudal middle frontal gyrus
Left isthmus cingulate cortex		Right inferior parietal cortex
Left middle temporal gyrus	*Structural Connectivity*	Right pars triangularis of inferior frontal gyrus
Left pars orbitalis of inferior frontal gyrus	Right lingual gyrus	
Left temporal pole	Right pars opercularis of inferior frontal gyrus	
Right banks of superior temporal sulcus	Right pars orbitalis of inferior frontal gyrus	
Right superior temporal gyrus		
Left pars orbitalis of inferior frontal gyrus		
Left temporal pole		
Right banks of superior temporal sulcus		
Right superior temporal gyrus		

Mega-analysis robustness and sensitivity analyses

Reproducibility of cortical hub vulnerability across different centrality metrics

To show that cortical hub vulnerability was reproducible across multiple network metrics of centrality, besides hub strength (degree centrality) we calculated the betweenness, eigenvector and closeness centralities of the 68x68 parcellated functional and structural cortical connectivity matrices of the HCP using R package "*NetworkToolbox*". All centrality metrics show a high degree of intercorrelation, (mean±SD: r_{func} = 0.69±0.31, r_{struc} = 0.9±0.08, Table S9). The high intercorrelation is somewhat expected, given that different aspects of the same underlying construct are measured. Similar to our main analysis using hub strength (degree centrality) (see main methods hub vulnerability model section), we examined the correlation between the cortical alteration map of SCZ with the betweenness, eigenvector and closeness centrality of each region. Correlations between SCZ-related cortical alterations and the additional centrality measures revealed very similar results as observed with hub strength (degree centrality) (Table S11).

Table S10. Correlation matrix of centrality measures

Functional Connectivity				
	Strength	Eigenvector	Betweenness	Closeness
Strength	1	1	0.41	0.98
Eigenvector	1	1	0.35	0.96
Betweenness	0.41	0.35	1	0.48
Closeness	0.98	0.96	0.48	1
**Structural Connectivity**				
	Strength	Eigenvector	Betweenness	Closeness
Strength	1	0.98	0.86	0.92
Eigenvector	0.98	1	0.83	0.95
Betweenness	0.86	0.83	1	0.79
Closeness	0.92	0.95	0.79	1

Table S11. Cortical hub vulnerability using different centrality metrics

Hub vulnerability	Centrality	Strength		Eigenvector		Betweenness		Closeness	
		R	p_{spin}	R	p_{spin}	R	p_{spin}	R	p_{spin}
Connectivity	Functional	0.58	<0.0001	0.54	0.0001	0.43	<0.0001	0.63	<0.0001
	Structural	0.32	0.02	0.26	0.04	0.36	0.01	0.27	0.04

Reproducibility across HCP age-matched and age-divergent ENIGMA SCZ samples

To show the robustness of our findings to the mean age group discrepancy of the multisite ENIGMA SCZ sample and the HCP sample, we split the ENIGMA SCZ sample into HCP age-matched groups and age-divergent groups. We first used the HCP mean age plus/minus 1SD to define an HCP age-matched ENIGMA SCZ sample (N = 1222, 495 SCZ) and an age-divergent group (N = 4084, 1944 SCZ). To show that the findings are robust across various definitions of

age-matched groups, we also used the HCP mean age plus/minus 2SDs to define the HCP age-matched ENIGMA SCZ sample (N = 2651, 1119 SCZ) and an age-divergent groups (N = 2655, N = 1320 SCZ). Regarding the matching to the HCP sample by age mean ± 1SD, we observed a very high agreement between the resulting t-values (r = 0.97, $p < 2.2e^{-16}$), and functional (r = 0.92, $p < 2.2e^{-16}$) and structural (r = 0.98, $p < 2.2e^{-16}$) epicenters. We are also able to confirm our finding for the hub vulnerability of functional (r = 0.49, $p_{spin} = 2e^{-05}$) and structural nodes (r = 0.26, $p_{spin} = 0.027$). We find similar results when matching the sample by age using the mean ± 2SD, observing an even higher agreement between the resulting t-values (r = 0.97, $p < 2.2e^{-16}$), and functional (r = 0.98, $p < 2.2e^{-16}$) and structural (r = 0.98, $p < 2.2e^{-16}$) epicenters. We are also able to confirm our finding for the preferential vulnerability of functional (r = 0.63, p= 1e-08) and structural nodes (r = 0.36, p = 0.003) to cortical thickness reduction. Lastly, we tested whether our results would be replicated in the most age-dissimilar group, i.e., individuals outside of the (mean+-2SD) window of the HCP distribution. Again, we could replicate our original findings robustly, with high agreement of t-values (r = 0.96, $p < 2.2e^{-16}$), and functional (r = 0.97, $p < 2.2e^{-16}$) and structural (r = 0.98, $p < 2.2e^{-16}$) epicenters. We are also able to confirm our finding for the preferential vulnerability of functional (r = 0.49, $p_{spin} = 2e^{-05}$) and structural hubs (r = 0.27, $p_{spin} = 0.023$).

Robustness and site-specific confirmation analysis of morphological alterations, hub vulnerability and disease epicenter models in SCZ

Cortical and subcortical alterations in SCZ

To examine the reproducibility of our mega-analytic findings and to make sure that our findings were not outlier-driven, we repeated our analysis within each participating site separately. One of the participating sites (GIPSI) was excluded from this site-specific analysis since this site contains data of patients only. In a first step, we computed the t-value map of the cortical thickness differences between patients and controls within each participating site. Site-specific schizophrenia-related cortical alterations were similar to our multisite mega-analytical findings (Fig S1a). In addition, in 21 out of 25 sites the spatial pattern of the schizophrenia-related cortical t-value map was significant correlated with the mega-analytic (multisite aggregation) cortical t-value map indicating good spatial similarity between the cortical alteration maps (Table S13).

Hub vulnerability and epicenter mapping

We next examined within each participating site the reproducibility of our finding that more central cortical nodes tend to display higher values of cortical thickness reductions. The

positive correlation of cortical thickness reduction with functional corticocortical degree centrality was more pronounced in functional networks (mean± SD: R= 0.23±0.28, Table S14) rather than structural networks (mean± SD: R= 0.15±0.14., Table S14), with 19 and 8 sites out of 25 showing statistical significance of this relationship respectively. In a final step, we examined the reproducibility of our epicenter findings, by identifying functional and structural epicenters of SCZ (see main methods for details) within each participating site. As observed in the multisite findings site-specific epicenters were most often identified in temporo-paralimbic extending to frontal brain regions (Fig S1b), with a high degree of correlation between our original map and the site-specific ones for functional, (median R=0.6; IQR=[0.26,0.8], Table S15) and structural epicenters (median R= 0.42; IQR=[0.25,0.55], Table S15)

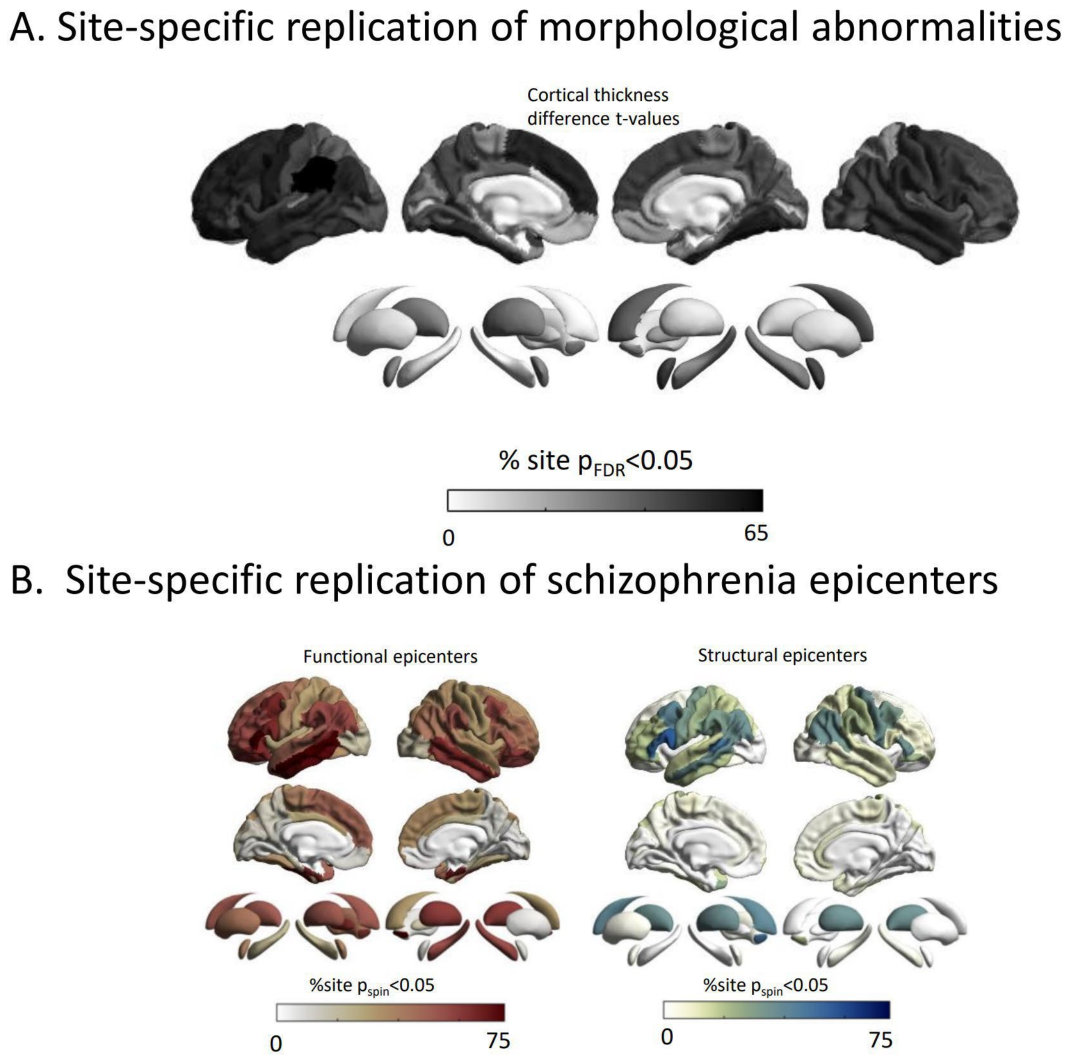

Figure S1. Site-specific replication of (A) morphological abnormalities in SCZ (B) functional and structural epicenters.

Table S13. Correlation between site-specific and mega-analytic cortical alteration maps of schizophrenia

Sites	R	p_{spin}
**ASRB**	0.53	<0.001
**fidmag**	0.58	<0.001
**FSL_Rome**	0.25	0.010
**IGP**	0.06	0.304
**Singapore**	0.58	<0.001
**Zurich**	0.33	0.005
**COBRE**	0.57	<0.001
**UCISZ**	0.67	<0.001
**PAFIP3T**	0.55	<0.001
**FOR210Marburg**	0.68	<0.001
**FOR210Muenster**	0.38	0.002
**SWIFT**	0.38	0.002
**CAMH**	0.36	0.002
**PAFIP1.5T**	0.64	<0.001
**STGO**	0.76	<0.001
**SCORE**	0.03	0.411
**UPenn**	0.48	<0.001
**PENS**	0.20	0.062
**PHCP**	0.21	0.028
**RSCZ_data**	0.73	<0.001
**MCIC**	0.76	<0.001
**ESO**	-0.35	0.999
**CIAM**	0.58	<0.001
**MPRC**	0.69	<0.001
**OLIN**	0.78	<0.001

Table S14. Site-specific hub vulnerability analysis

Sites	R_{func}	p_{func}	R_{struc}	p_{struc}
**ASRB**	-0.13	0.644	0.13	0.194
**fidmag**	0.39	0.006	0.04	0.367
**FSL_Rome**	0.60	0.010	0.40	0.001
**IGP**	-0.31	0.865	-0.05	0.641
**Singapore**	0.53	<0.001	0.06	0.316
**Zurich**	-0.14	0.695	-0.14	0.828
**COBRE**	-0.05	0.575	0.19	0.099
**UCISZ**	0.21	0.081	0.22	0.045
**PAFIP3T**	0.64	0.001	0.30	0.017
**FOR210Marburg**	0.42	0.002	0.23	0.041
**FOR210Muenster**	-0.04	0.566	0.16	0.118
**SWIFT**	0.24	0.059	0.21	0.066
**CAMH**	0.34	0.073	0.17	0.124
**PAFIP1.5T**	0.28	0.029	0.16	0.104
**STGO**	0.62	<0.001	0.23	0.053
**SCORE**	0.22	0.069	-0.01	0.530
**UPenn**	0.22	0.097	0.12	0.179
**PENS**	0.13	0.191	0.22	0.052
**PHCP**	0.46	0.031	-0.18	0.915
**RSCZ_data**	0.40	0.011	0.45	0.001
**MCIC**	0.34	0.012	0.20	0.066
**ESO**	-0.35	0.953	0.12	0.187
**CIAM**	0.05	0.390	0.08	0.262

**MPRC**	0.55	0.002	0.20	0.079
**OLIN**	0.24	0.103	0.15	0.141

Table S15. Site-specific epicenter map agreement with mega-analytical epicenter map

Sites	R_{func}	p_{func}	R_{struc}	p_{struc}
**ASRB**	0.48	0	0.48	0
**fidmag**	0.8	0	0.44	0
**FSL_Rome**	0.17	0.132	0.1	0.124
**IGP**	0.15	0.132	0.25	0.115
**Singapore**	0.82	0	0.55	0
**Zurich**	0.45	0	0.31	0
**COBRE**	0.63	0	0.48	0
**UCISZ**	0.87	0	0.71	0
**PAFIP3T**	0.52	0	0.33	0
**FOR210Marburg**	0.77	0	0.54	0
**FOR210Muenster**	0.57	0	0.32	0
**SWIFT**	0.55	0	0.47	0
**CAMH**	-0.03	0.587	-0.25	0.598
**PAFIP1.5T**	0.83	0	0.56	0
**STGO**	0.86	0	0.56	0
**SCORE**	-0.12	0.832	-0.07	0.845
**UPenn**	0.88	0	0.69	0
**PENS**	-0.02	0.557	-0.19	0.567
**PHCP**	0.26	0.016	-0.08	0.007
**RSCZ_data**	0.64	0	0.41	0
**MCIC**	0.92	0	0.68	0
**ESO**	-0.55	1	-0.42	1
**CIAM**	0.77	0	0.41	0
**MPRC**	0.6	0	0.42	0
**OLIN**	0.8	0	0.55	0

Subject-level cortical abnormality modeling

We next sought to examine whether our network-based models can be translated to individual schizophrenia patients' data and how they are influenced by individual clinical factors. Batch-corrected cortical thickness data of patients were first adjusted for age and sex by residualizing the effect of age and sex using a linear model. Subsequently they were z-scored relative to healthy controls to generate individualized morphological abnormality z-score maps. To test the hub vulnerability hypothesis at an individual level we descriptively compared the expected vs observed incidence of statistically significant positive correlations of nodal centrality and patient-specific morphological abnormality maps, adjusting for spatial autocorrelation using spin permutation tests. According to an alpha of 0.05 and the properties of the Gaussian distribution we define the expected incidence of statistically significant positive correlation values as 0.025. We next sought to test the robustness of our epicenter findings at the individual patient level. We identified patient-specific structural and functional epicenter maps by iteratively correlating the connectivity profile of each brain region to each patient's morphological abnormality map as described in the epicenter mapping section. Significance

was tested for each patient-specific epicenters using spin permutation test (p_{spin}<0.05) as described in the method section in the manuscript. We finally computed the percentage of individuals for which each region was a statistically significant epicenter, correcting for multiple testing with the Bonferroni method.

Subject-level hub vulnerability modeling

To assess whether network atrophy models can also explain individual patient data, each patient-specific cortical abnormality map was correlated with the normative degree centrality maps (Fig. S2). We observed similar associations between individual cortical maps and functional (p_{spin} < 0.05 in 18.2% of individuals with SCZ) as well as structural cortico-cortical hubs (p_{spin} < 0.05 in 8.1% of individuals with SCZ) as seen in the group-level analysis. By contrast, a null distribution of p-values (corrected for spatial autocorrelation) would only show a rate of approximately 2,5% statistically significant positive correlations. Thus, we observed a 7.2-fold (18.2% vs. 2.5%; $\chi^2_{func} = 2466.4$, df = 1, p-value < $2.2e^{-16}$) and 3.2-fold (8.1% vs 2.5%, $\chi^2_{struc} = 315.58$, df = 1, p-value < $2.2e^{-16}$) enrichment of significant associations between individual morphometric maps and cortical centrality maps than would be expected in the null hypothesis.

Subject-level epicenter modeling

Using each patient's individual cortical abnormality map, we further identified patient-specific structural and functional epicenters with a marked overlap in the top significant epicenters identified by our mega-analysis. Specifically, 9 out of 10 top epicenters overlap between the individual epicenter models and our group-level mega-analysis including the entorhinal cortices, banks of superior temporal sulci, left inferior temporal gyrus, and frontal gyri (bilateral pars triangularis, left pars opercularis, right pars orbitalis) (Fig. S2). In summary, although the individual subject-level data displayed overall lower sensitivity, due to the increased heterogeneity in cortical abnormality patterns, the results closely mirrored our group-level analysis. Collectively, individual network modeling supported both the hub vulnerability hypothesis and the most significant epicenters as identified by our mega-analysis.

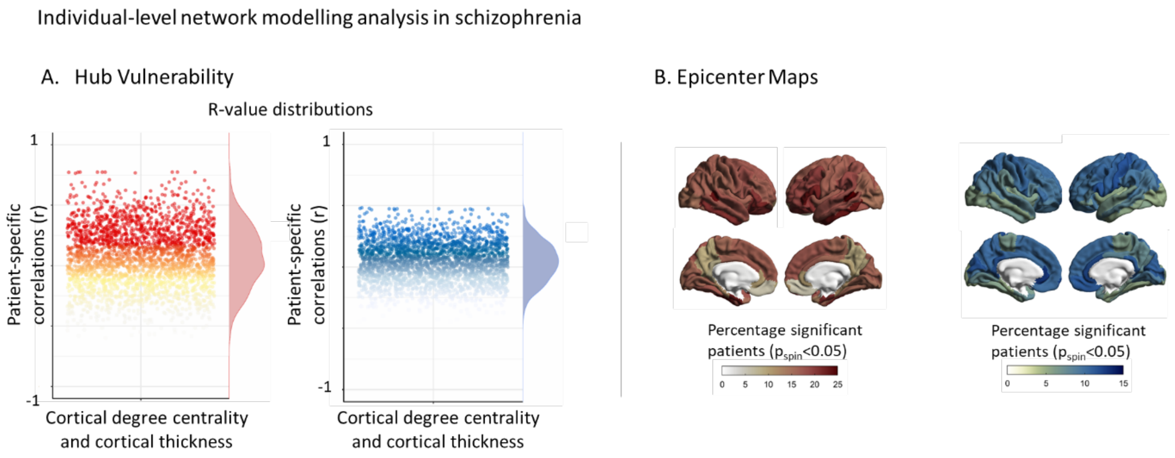

Figure S2. Individual-level network modeling analysis in schizophrenia. (A) Hub vulnerability. On an individual patient level, we computed patient-specific morphological abnormality maps and tested the hub vulnerability hypothesis for each patient correcting for spatial autocorrelation (pspin<0.05). The resulting R-value distributions are enriched in positive correlations (7.2-fold, 18.2% vs. 2.5%; χ^2_{func} = 2466.4, df = 1, p-value < 2.2e^{-16} and 3.2-fold, 8.1% vs 2.5%, χ^2 $=3_{st}1_{ru}5_{c}$ .58, df = 1, p-value < 2.2e^{-16}). (B) Epicenter Mapping. On an individual patient level, we computed patient-specific disease epicenters by identifying regions with a connectivity profile which significantly correlated (p_{spin}<0.05) with each patient's morphological abnormality map. Epicenter map depicts the percentage of individual patients for whom each region is a significant functional (above) or structural (below) epicenter. The ranking of regions most highly enriched for statistical significance in individuals correlates highly with the original epicenter map.

Subject-level correlation of clinical variables to hub vulnerability and epicenters

Having established both network-based models in subject-level data (see results above), we next examined the association between individual clinical factors and patient's hub vulnerability and epicenters respectively. To this end, we correlated the subject-level hub vulnerability scores with antipsychotic medication, duration of illness, PANSS total, PANSS positive, negative and general scores (Table S16). To examine the relationship between individual subject-level epicenters and clinical factors, we correlated each patient's epicenters (based on significance) with the above-mentioned clinical factors. To control for multiple comparisons p-values were adjusted within each clinical variable analysis using the Bonferroni method. (Tables S17-18).

Table S16. Correlations between subject-level functional and structural hub vulnerability and clinical scores

**Individual Hub Vulnerability**				
	**Functional Connectivity**		**Structural Connectivity**	
Clinical Variables	R	pval (Bonferroni)	R	pval (Bonferroni)
PANSS Positive	0.06	0.027	0.06	0.025
PANSS Negative	0.03	0.241	0.05	0.018
PANSS General	0.21	<0.0001	0.13	0.01
PANSS Total	0.1	0.001	0.09	0.004
Chlorpromazine	-0.02	0.403	-0.01	0.832
Duration of Illness	-0.04	0.112	0.02	0.309
All p-values are corrected for multiple comparison using the Bonferroni method				

Table S17. Correlations between individual subject-level functional epicenters and clinical scores

**Functional connectivity**												
	PANSS Positive		PANSS Negative		PANSS General		PANSS Total		Chlorpromazine equivalents		Duration of Illness	
**Brain Region**	R	pval	R	pval	R	pval	R	pval	R	pval	R	pval
Left banks of superior temporal sulcus	0.05	1	0.03	1	0.15	0.296	0.09	0.296	0.02	1	-0.04	1
Left caudal anterior cingulate cortex	0.05	1	0.04	1	0.24	<0.001	0.1	0.088	-0.03	1	-0.03	1
Left caudal middle frontal gyrus	0.04	1	0.02	1	0.06	1	0.06	1	0.01	1	-0.02	1
Left cuneus	0.04	1	0.01	1	0.22	0.001	0.08	0.704	-0.03	1	-0.03	1
Left entorhinal cortex	0.04	1	0.03	1	0.07	1	0.08	0.981	0	1	-0.01	1
Left fusiform gyrus	0.05	1	0.01	1	0.17	0.052	0.08	0.763	-0.03	1	-0.02	1
Left inferior parietal cortex	0.04	1	0	1	0.06	1	0.05	1	0	1	-0.04	1
Left inferior temporal gyrus	0.06	0.929	0.02	1	0.18	0.019	0.1	0.121	0.01	1	-0.02	1
Left isthmus cingulate cortex	0.04	1	0.01	1	0.11	1	0.05	1	-0.03	1	-0.01	1
Left lateral occipital cortex	0.02	1	-0.01	1	0.15	0.249	0.05	1	-0.04	1	-0.03	1
Left lateral orbitofrontal cortex	0.03	1	0.02	1	0.11	1	0.06	1	0.02	1	-0.03	1
Left lingual gyrus	0.04	1	0.02	1	0.23	<0.001	0.09	0.299	-0.05	1	-0.02	1
Left medial orbitofrontal cortex	0.01	1	0.01	1	-0.01	1	0.02	1	0	1	0.02	1

Left middle temporal gyrus	0.05	1	0.02	1	0.02	1	0.05	1	0.04	1	-0.01	1
Left parahippocampal gyrus	0.04	1	0.03	1	0.13	0.957	0.07	1	-0.03	1	0	1
Left paracentral lobule	0.08	0.088	0.05	1	0.24	<0.001	0.13	0.002	-0.02	1	-0.04	1
Left pars opercularis of inferior frontal gyrus	0.04	1	0.01	1	0.15	0.284	0.07	1	0.01	1	-0.05	0.979
Left pars orbitalis of inferior frontal gyrus	-0.01	1	0	1	-0.05	1	0	1	0.03	1	-0.04	1
Left pars triangularis of inferior frontal gyrus	0.04	1	0.02	1	0.09	1	0.06	1	0.06	1	-0.03	1
Left pericalcarine cortex	0.03	1	0.02	1	0.22	0.001	0.07	1	-0.06	1	-0.01	1
Left postcentral gyrus	0.09	0.022	0.05	1	0.26	<0.001	0.14	<0.001	-0.01	1	-0.03	1
Left posterior cingulate cortex	0.04	1	0.03	1	0.2	0.005	0.09	0.308	-0.03	1	-0.03	1
Left precentral gyrus	0.08	0.143	0.05	1	0.24	<0.001	0.13	0.002	-0.01	1	-0.04	1
Left precuneus	0.05	1	0.02	1	0.18	0.025	0.09	0.204	-0.05	1	-0.01	1
Left rostral anterior cingulate cortex	0.03	1	0.04	1	0.11	1	0.06	1	-0.04	1	-0.01	1
Left rostral middle frontal gyrus	0.04	1	0.02	1	0.16	0.126	0.08	0.691	0	1	-0.02	1
Left superior frontal gyrus	0.05	1	0.02	1	0.14	0.342	0.08	0.791	0	1	-0.03	1
Left superior parietal cortex	0.05	1	0.03	1	0.23	0.001	0.1	0.073	-0.04	1	-0.02	1

Left superior temporal gyrus	0.05	1	0.03	1	0.18	0.035	0.1	0.103	-0.01	1	-0.05	1
Left supramarginal gyrus	0.05	1	0.02	1	0.13	0.68	0.07	1	-0.01	1	-0.04	1
Left frontal pole	0	1	0.01	1	-0.08	1	0	1	0.04	1	0	1
Left temporal pole	-0.01	1	-0.01	1	-0.14	0.442	-0.03	1	0.07	1	-0.02	1
Left transverse temporal gyrus	0.07	0.56	0.04	1	0.24	<0.001	0.11	0.014	-0.01	1	-0.02	1
Left insula	0.05	1	0.03	1	0.23	<0.001	0.1	0.059	-0.02	1	-0.04	1
Right banks of superior temporal sulcus	0.04	1	0.03	1	0.16	0.081	0.09	0.265	0.01	1	-0.05	1
Right caudal anterior cingulate cortex	0.05	1	0.03	1	0.21	0.003	0.1	0.071	-0.03	1	-0.03	1
Right caudal middle frontal gyrus	0.01	1	-0.02	1	0.05	1	0.03	1	-0.02	1	-0.05	1
Right cuneus	0.05	1	0.03	1	0.24	<0.001	0.1	0.057	-0.06	1	-0.02	1
Right entorhinal cortex	0.04	1	0.01	1	0.03	1	0.05	1	0.02	1	-0.02	1
Right fusiform gyrus	0.04	1	0.02	1	0.15	0.191	0.07	1	-0.04	1	-0.01	1
Right inferior parietal cortex	0.04	1	0.01	1	0.13	0.895	0.07	1	-0.03	1	-0.03	1
Right inferior temporal gyrus	0.03	1	0	1	0.08	1	0.05	1	-0.01	1	-0.04	1
Right isthmus cingulate cortex	0.03	1	0.01	1	0.13	0.682	0.06	1	-0.07	1	-0.02	1
Right lateral occipital cortex	0.04	1	0.03	1	0.18	0.025	0.08	0.753	-0.03	1	-0.01	1
Right lateral orbitofrontal cortex	0.02	1	-0.01	1	0.1	1	0.03	1	0.01	1	-0.05	1
Right lingual gyrus	0.04	1	0.01	1	0.21	0.002	0.07	1	-0.05	1	-0.02	1

Right medial orbitofrontal cortex	-0.01	1	0.01	1	-0.06	1	0	1	0	1	0.01	1
Right middle temporal gyrus	0.03	1	0	1	0.06	1	0.04	1	0.02	1	-0.01	1
Right parahippocampal gyrus	0.03	1	0.02	1	0.14	0.42	0.08	0.832	-0.04	1	-0.01	1
Right paracentral lobule	0.06	0.724	0.04	1	0.24	<0.001	0.12	0.01	-0.02	1	-0.03	1
Right pars opercularis of inferior frontal gyrus	0.04	1	0.03	1	0.17	0.044	0.09	0.378	-0.01	1	-0.03	1
Right pars orbitalis of inferior frontal gyrus	0.01	1	0.01	1	-0.04	1	0.01	1	0.04	1	-0.01	1
Right pars triangularis of inferior frontal gyrus	0.02	1	0	1	0.12	1	0.04	1	0.01	1	-0.06	0.443
Right pericalcarine cortex	0.04	1	0	1	0.22	0.001	0.07	1	-0.05	1	-0.03	1
Right postcentral gyrus	0.09	0.041	0.06	0.877	0.24	<0.001	0.14	<0.001	-0.01	1	-0.03	1
Right posterior cingulate cortex	0.04	1	0.03	1	0.21	0.002	0.09	0.306	-0.03	1	-0.03	1
Right precentral gyrus	0.06	1	0.03	1	0.21	0.001	0.1	0.064	-0.01	1	-0.03	1
Right precuneus	0.04	1	0.03	1	0.19	0.011	0.09	0.325	-0.06	1	0	1
Right rostral anterior cingulate cortex	-0.03	1	0.02	1	0.06	1	0.01	1	-0.03	1	0.01	1
Right rostral middle frontal gyrus	0.03	1	0.01	1	0.14	0.303	0.05	1	-0.02	1	-0.04	1

Right superior frontal gyrus	0.04	1	0.02	1	0.16	0.088	0.08	0.455	-0.02	1	-0.05	1
Right superior parietal cortex	0.05	1	0.02	1	0.18	0.02	0.08	0.762	-0.03	1	-0.02	1
Right superior temporal gyrus	0.05	1	0.03	1	0.17	0.057	0.09	0.34	-0.01	1	-0.04	1
Right supramarginal gyrus	0.07	0.57	0.03	1	0.23	<0.001	0.11	0.015	-0.01	1	-0.03	1
Right frontal pole	-0.01	1	0.01	1	-0.08	1	-0.02	1	0.02	1	-0.01	1
Right temporal pole	-0.01	1	0.01	1	-0.13	0.83	-0.01	1	0.03	1	-0.01	1
Right transverse temporal gyrus	0.06	0.82	0.05	1	0.26	<0.001	0.12	0.008	-0.02	1	-0.04	1
Right insula	0.05	1	0.04	1	0.24	<0.001	0.11	0.033	-0.02	1	-0.04	1
All p-values are corrected for multiple comparison using the Bonferroni method												

Table S18. Correlations between individual subject-level structural epicenters and clinical scores

Structural Connectivity												
	PANSS Positive		PANSS Negative		PANSS General		PANSS Total		Chlorpromazine equivalents		Duration of Illness	
	R	pval	R	pval	R	pval	R	pval	R	pval	R	pval
Left banks of superior temporal sulcus	-0.01	1	0.01	1	-0.09	1	0	1	0.02	1	0.01	1
Left caudal anterior cingulate cortex	-0.01	1	0.02	1	0.04	1	-0.01	1	-0.01	1	0.02	1
Left caudal middle frontal gyrus	0.08	0.228	0.07	0.009	0.1	0.265	0.11	0.001	0.02	1	0	
Left cuneus	0.01	1	-0.01	1	0.11	1	0.03	1	-0.06	1	0.01	1
Left entorhinal cortex	-0.01	1	0.01	1	-0.12	1	-0.01	1	-0.04	1	0.03	1
Left fusiform gyrus	0	1	-0.02	1	-0.08	1	-0.02	1	-0.04	1	0.03	0.415
Left inferior parietal cortex	0.03	1	0.03	1	-0.04	1	0.04	1	0.02	1	0.01	1
Left inferior temporal gyrus	0.01	1	0.01	1	-0.03	1	0.02	1	-0.01	1	0.02	1
Left isthmus cingulate cortex	0.02	1	-0.02	1	0.11	1	0.02	1	-0.05	1	0.03	1
Left lateral occipital cortex	0.03	0.065	-0.02	1	-0.11	1	-0.03	0.143	-0.05	1	0.03	1
Left lateral orbitofrontal cortex	0.02	1	0.03	1	-0.04	1	-0.02	1	0.04	1	0.04	1
Left lingual gyrus	0.02	1	-0.02	1	-0.04	1	-0.03	1	-0.05	1	0.01	1
Left medial orbitofrontal cortex	0.05	1	0	1	-0.13	1	-0.06	1	0.03	1	0.04	1
Left middle temporal gyrus	0.02	1	0.01	0.508	-0.07	1	0.01	1	0.04	1	0.03	1
Left parahippocampal gyrus	0.02	1	-0.01	1	-0.08	1	-0.03	1	-0.05	1	0.02	1
Left paracentral lobule	0.12	1	0.07	1	0.24	1	0.16	1	-0.02	1	0	1
Left pars opercularis of inferior frontal gyrus	0.04	1	0.03	1	0.04	1	0.05	1	0.05	1	0	1

Left pars orbitalis of inferior frontal gyrus	0.01	1	0.01	0.386	-0.01	1	0	1	0.05	1	-0.01	1
Left pars triangularis of inferior frontal gyrus	0.02	1	0.03	1	-0.04	1	0.02	1	0.08	1	0.01	1
Left pericalcarine cortex	0.01	1	-0.01	1	0.01	1	-0.01	1	-0.06	1	0.02	0.134
Left postcentral gyrus	0.11	1	0.06	1	0.21	1	0.15	1	0	1	-0.02	0.154
Left posterior cingulate cortex	0.03	1	0.04	1	0.11	1	0.06	1	0.01	1	0.05	1
Left precentral gyrus	0.15	1	0.08	1	0.23	0.064	0.17	0.357	0.04	1	-0.01	1
Left precuneus	0.06	1	0.02	1	0.17	1	0.08	1	-0.04	1	0.01	1
Left rostral anterior cingulate cortex	0.01	1	0.03	1	-0.01	1	-0.01	1	0.01	0.533	0.02	1
Left rostral middle frontal gyrus	0.01	1	0.02	1	-0.01	1	0.01	1	0.04	1	0.01	1
Left superior frontal gyrus	0.06	1	0.04	1	0.13	0.659	0.06	1	0.01	1	0.03	1
Left superior parietal cortex	0.05	1	0.04	1	0.23	1	0.11	1	-0.07	1	0	1
Left superior temporal gyrus	0.02	1	0.01	1	-0.06	1	-0.01	1	0.02	1	0	1
Left supramarginal gyrus	0.02	1	0.01	1	-0.01	1	0.03	1	0.04	1	-0.03	1
Left frontal pole	0.01	1	0.02	1	-0.01	1	-0.01	1	0.02	1	0.01	1
Left temporal pole	0.08	0.118	-0.02	1	-0.29	0.065	-0.12	0.346	0.05	1	0.02	1
Left transverse temporal gyrus	0.04	1	0.04	1	0.05	1	0.06	1	0.03	1	0	1
Left insula	0	1	0.03	1	-0.04	1	0	1	0	1	0.02	1
Right banks of superior temporal sulcus	0.04	1	-0.01	1	-0.15	1	-0.05	1	0.01	1	-0.03	1
Right caudal anterior cingulate cortex	0.01	1	0.02	1	0.02	1	0.01	1	0.01	1	0.02	1
Right caudal middle frontal gyrus	0.07	1	0.05	1	0.19	0.166	0.11	1	0.01	1	-0.02	0.185
Right cuneus	0.01	1	-0.01	1	0.09	1	0.03	1	-0.06	1	0.01	1
Right entorhinal cortex	0	1	-0.01	1	-0.08	1	-0.02	1	0.03	1	0.03	1
Right fusiform gyrus	0.01	1	-0.02	1	-0.04	1	-0.02	1	-0.01	1	0.02	1

Right inferior parietal cortex	0.02	1	-0.01	1	0.02	1	0.01	1	0.01	1	-0.04	1
Right inferior temporal gyrus	0.04	1	-0.03	1	-0.16	1	-0.08	1	0.03	1	0	1
Right isthmus cingulate cortex	0	1	0	1	0.1	1	0.02	1	-0.08	1	0.03	1
Right lateral occipital cortex	0.02	1	-0.02	1	-0.01	1	-0.02	1	-0.01	1	0.01	1
Right lateral orbitofrontal cortex	0.05	1	-0.02	1	-0.08	1	-0.07	1	0.03	1	-0.01	1
Right lingual gyrus	0.02	1	-0.02	1	-0.01	0.558	-0.02	1	-0.04	1	0.03	1
Right medial orbitofrontal cortex	0.03	1	0	1	-0.05	1	-0.04	1	0.02	0.164	0.02	1
Right middle temporal gyrus	0.03	1	-0.01	1	-0.03	1	-0.03	1	0.02	1	0	1
Right parahippocampal gyrus	0.04	1	-0.01	1	-0.04	1	-0.03	1	-0.01	1	0.03	0.759
Right paracentral lobule	0.1	1	0.07	1	0.26	1	0.16	1	-0.03	1	0	1
Right pars opercularis of inferior frontal gyrus	0.05	0.865	0.04	1	0.17	1	0.08	0.958	0	1	-0.03	1
Right pars orbitalis of inferior frontal gyrus	0.05	1	-0.02	1	-0.13	1	-0.06	1	-0.01	1	-0.02	1
Right pars triangularis of inferior frontal gyrus	0.01	1	0.01	1	0.04	1	0.02	1	0.01	1	-0.06	1
Right pericalcarine cortex	0.01	1	-0.01	1	0.1	1	0.01	1	-0.06	1	0	1
Right postcentral gyrus	0.09	0.502	0.05	1	0.16	0.735	0.11	1	-0.01	1	-0.01	1
Right posterior cingulate cortex	0.08	1	0.06	1	0.23	1	0.12	1	-0.01	1	0.02	1
Right precentral gyrus	0.1	0.504	0.05	1	0.22	0.066	0.13	1	0	1	-0.03	0.574
Right precuneus	0.04	1	0.03	1	0.16	1	0.08	1	-0.04	1	0.03	1
Right rostral anterior cingulate cortex	0.05	1	0	1	-0.04	1	-0.05	1	0.02	1	0.02	1
Right rostral middle frontal gyrus	0	1	0	1	0.05	1	-0.01	1	0.02	1	-0.03	1
Right superior frontal gyrus	0.06	1	0.04	1	0.14	1	0.07	1	0.01	1	0.02	1

Right superior parietal cortex	0.03	1	0.01	1	0.13	1	0.06	1	-0.05	1	-0.02	1
Right superior temporal gyrus	0.08	1	-0.05	1	-0.22	1	-0.11	1	0.02	1	-0.02	1
Right supramarginal gyrus	0	1	-0.02	1	-0.02	1	0	1	0.01	1	-0.05	1
Right frontal pole	0.01	1	0.02	1	0.03	1	0	1	0.03	1	0	1
Right temporal pole	0.04	1	0	1	-0.17	1	-0.06	1	0.01	1	0.02	1
Right transverse temporal gyrus	0	1	0.01	1	-0.04	1	0	1	0.02	1	-0.01	1
Right insula	0.03	1	0.01	1	0.01	1	-0.01	1	0.01	1	-0.02	1
All p-values are corrected for multiple comparison using the Bonferroni method												

Subject-level robustness and sensitivity analysis

To show the robustness of the correlation values between clinical variables and our individual-level hub vulnerability and epicenter findings we performed a sensitivity permutation analysis in our sample. Specifically, we generated 100 different permutations of 80% of our sample- i.e., an 80-20 split without resampling. We accordingly repeated the correlation analysis with the clinical variables 100 times. Our results show that the original findings are highly robust to perturbation of our sample (Fig S3-9).

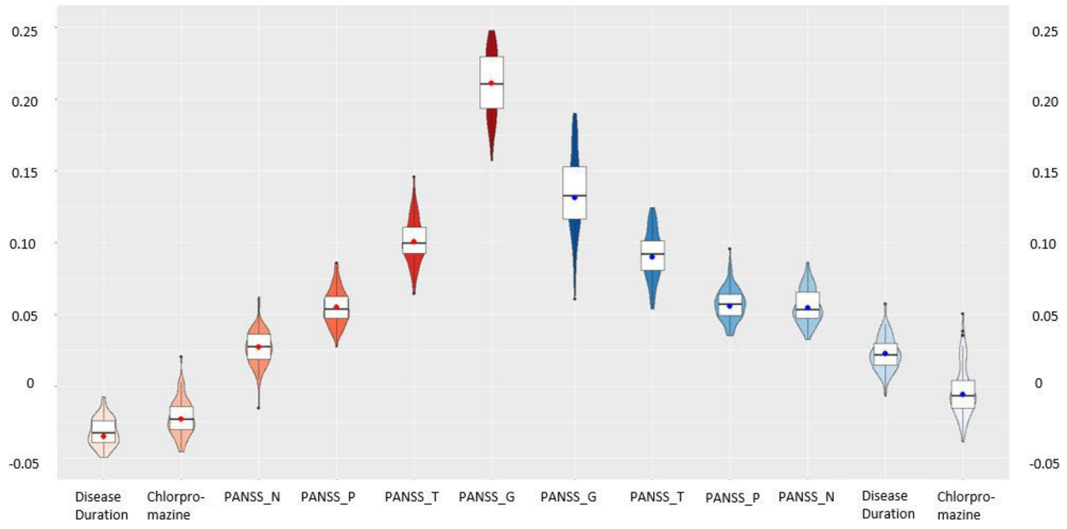

Figure S3. Robustness-Permutation analysis of stability of correlation of hub vulnerability at the individual level to individual clinical symptoms. Violin plots represent the distribution of values of the 100 permutations using a different 80% of the sample. Central dot represents the value obtained using 100% of our sample.

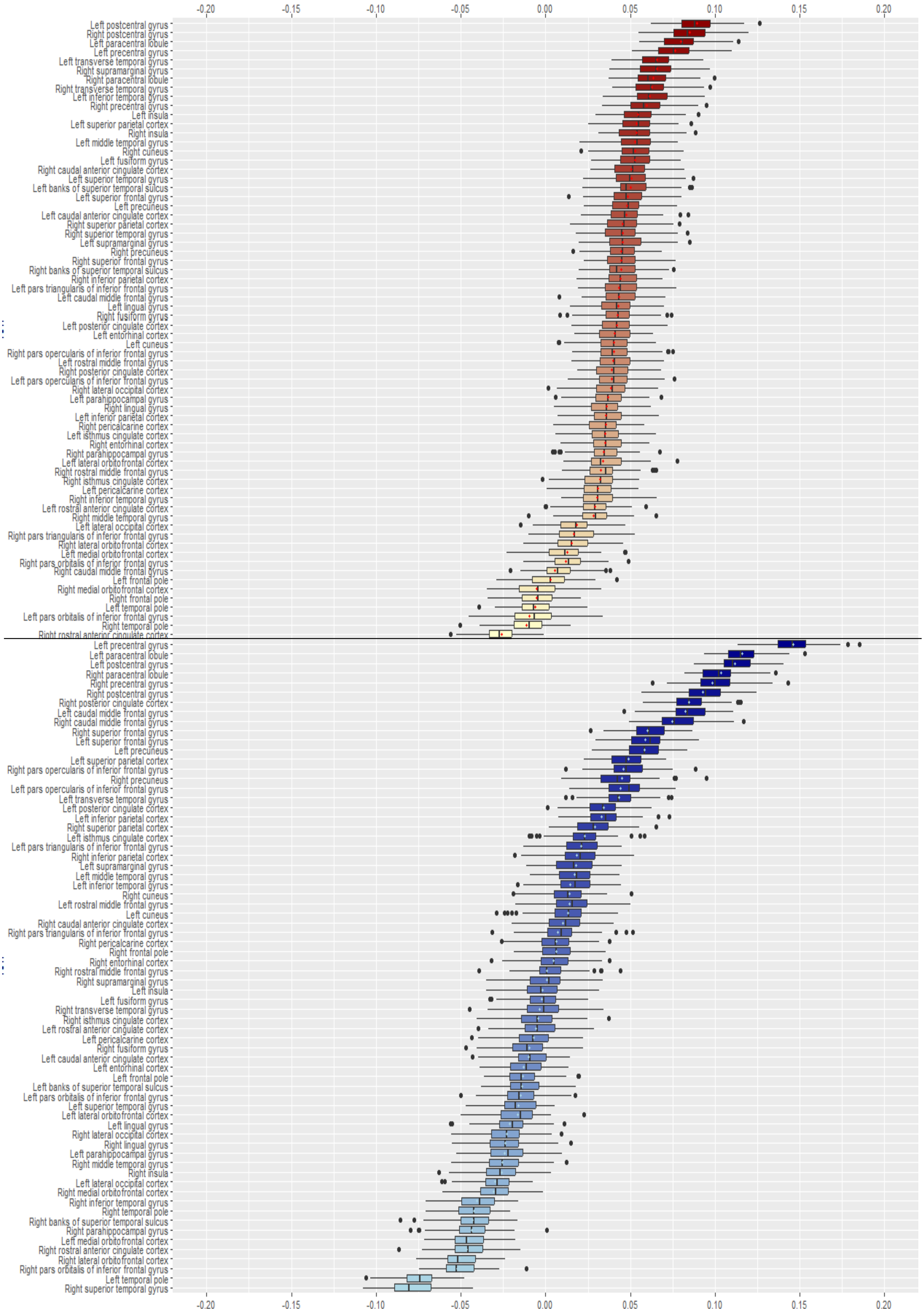

Figure S4. Robustness-Permutation analysis of stability of correlation of epicenter values at the individual level to individual PANSS Positive symptoms. Boxplots represent the distribution of values of the 100 permutations using a different 80% of the sample. Central dot represents the value obtained using 100% of our sample.

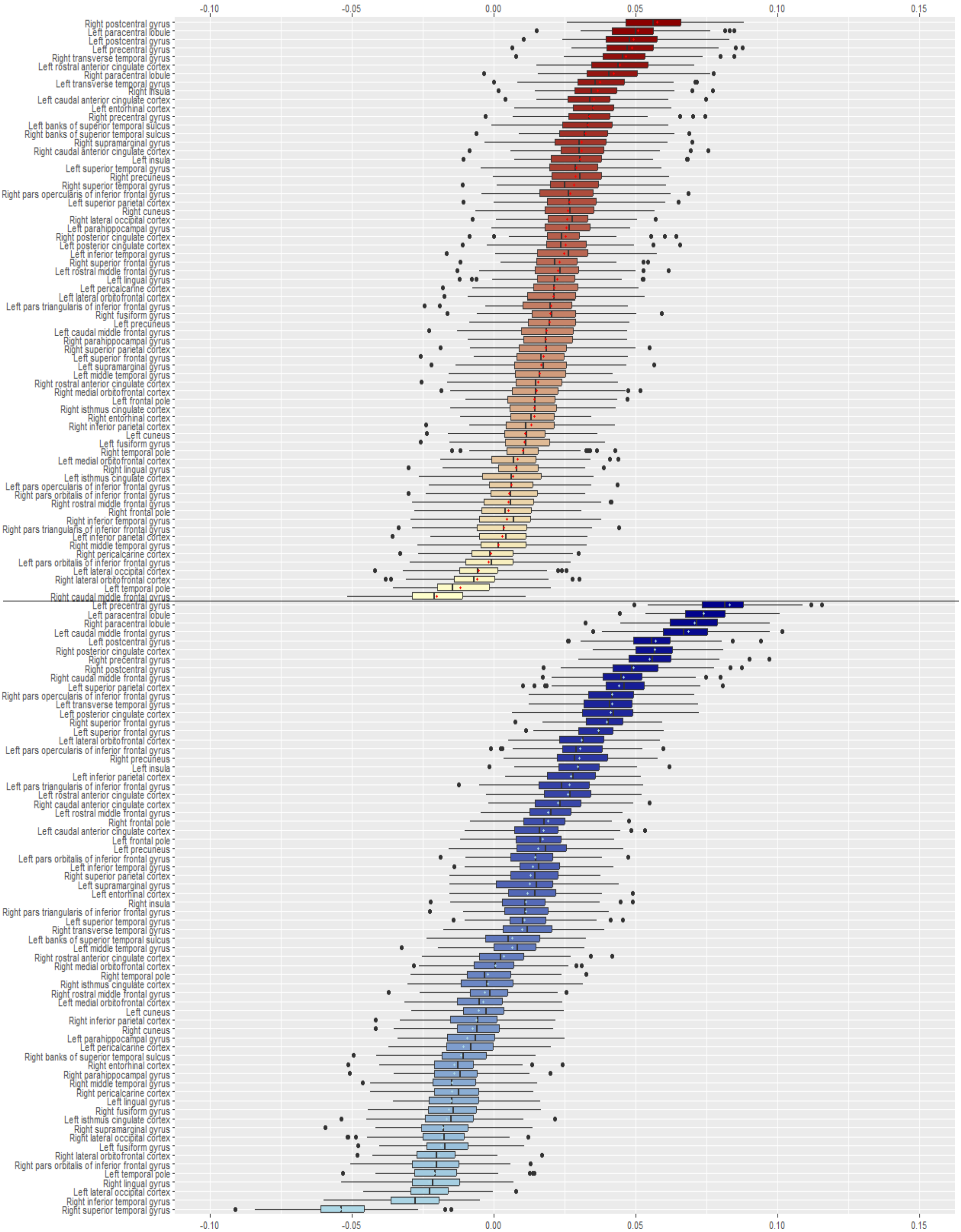

Figure S5. Robustness-Permutation analysis of stability of correlation of epicenter values at the individual level to individual PANSS Negative symptoms. Boxplots represent the distribution of values of the 100 permutations using a different 80% of the sample. Central dot represents the value obtained using 100% of our sample.

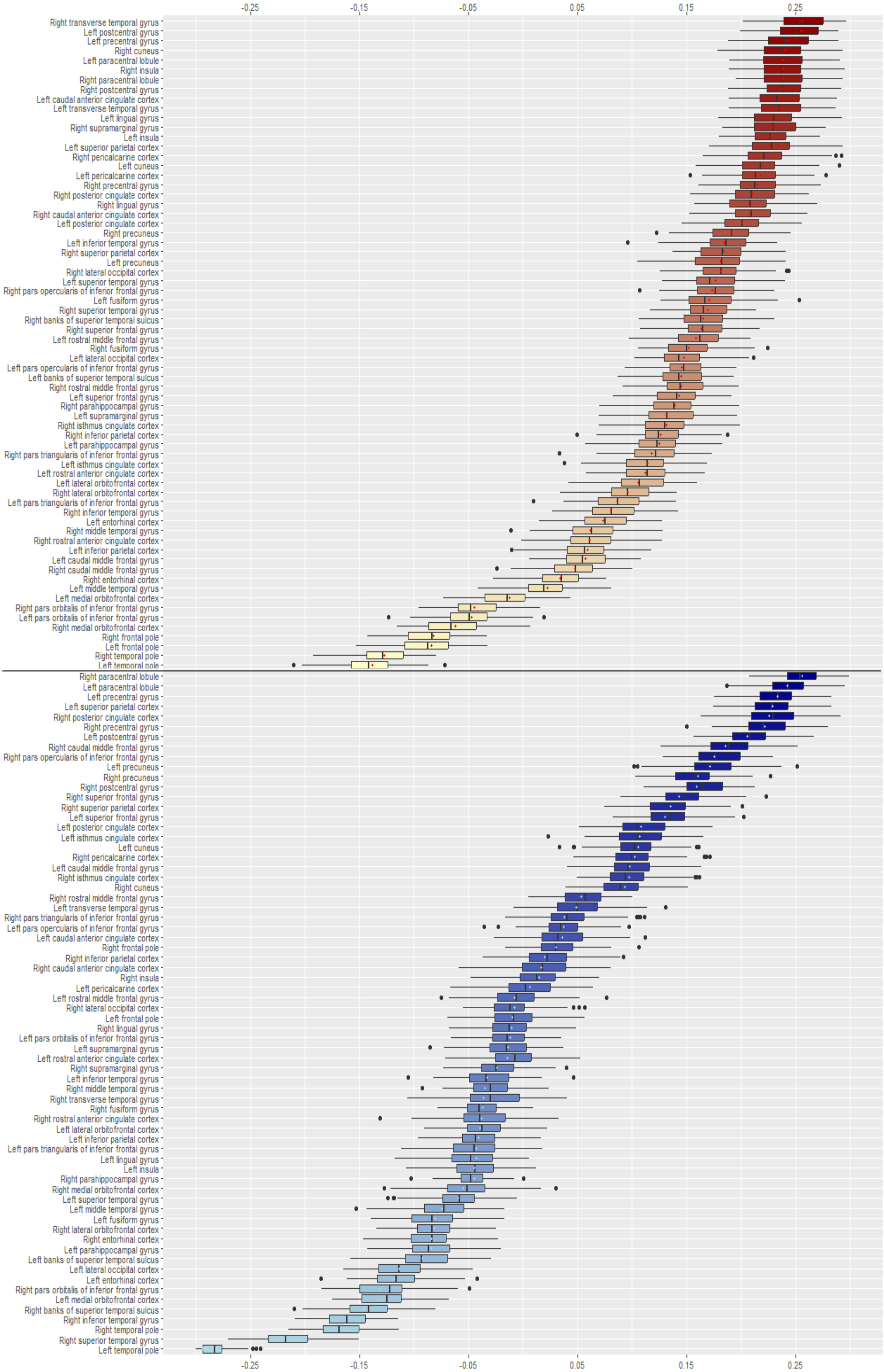

Figure S6. Robustness-Permutation analysis of stability of correlation of epicenter values at the individual level to individual PANSS General symptoms. Boxplots represent the distribution of values of the 100 permutations using a different 80% of the sample. Central dot represents the value obtained using 100% of our sample.

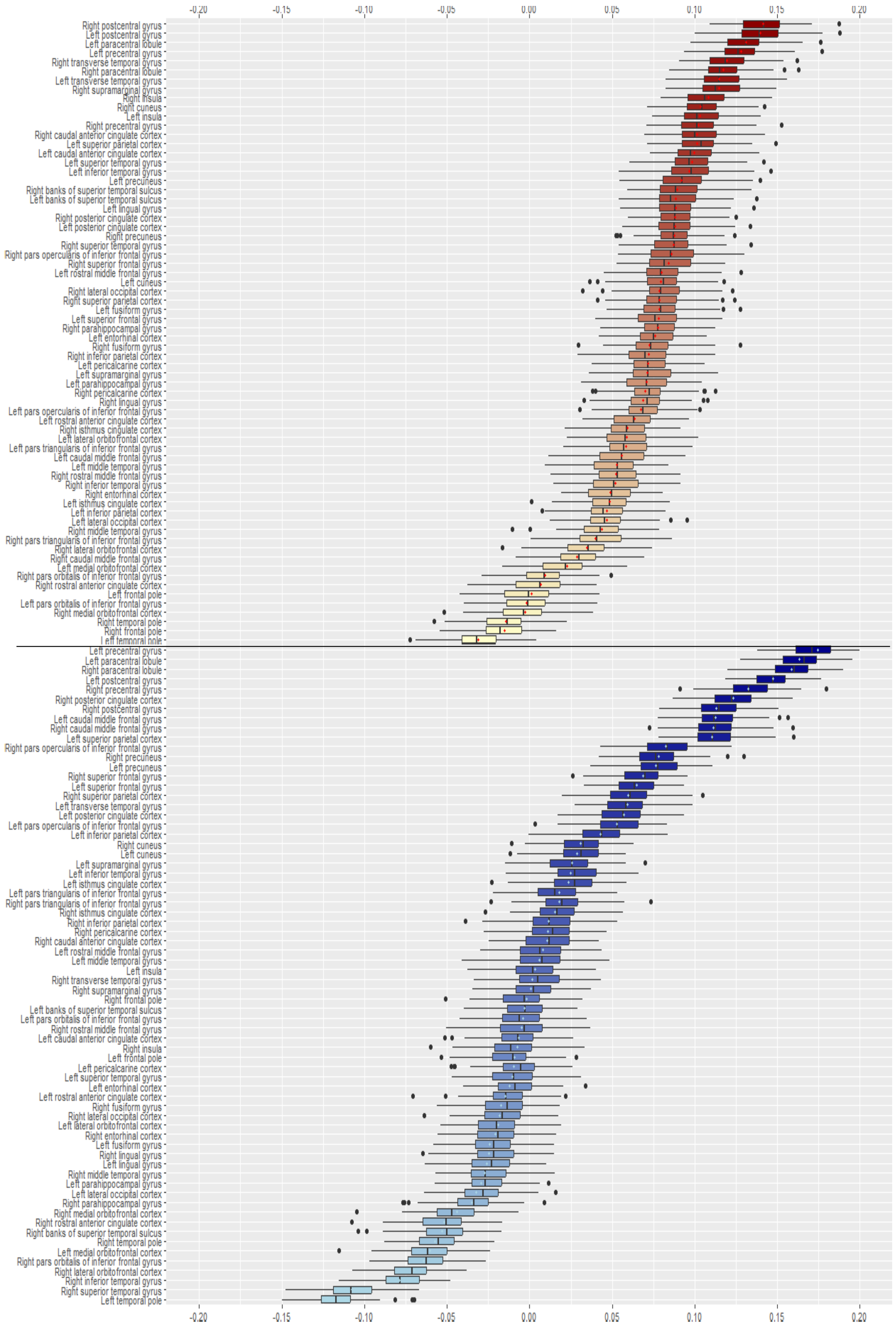

Figure S7. Robustness-Permutation analysis of stability of correlation of epicenter values at the individual level to individual PANSS Total Score. Boxplots represent the distribution of values of the 100 permutations using a different 80% of the sample. Central dot represents the value obtained using 100% of our sample.

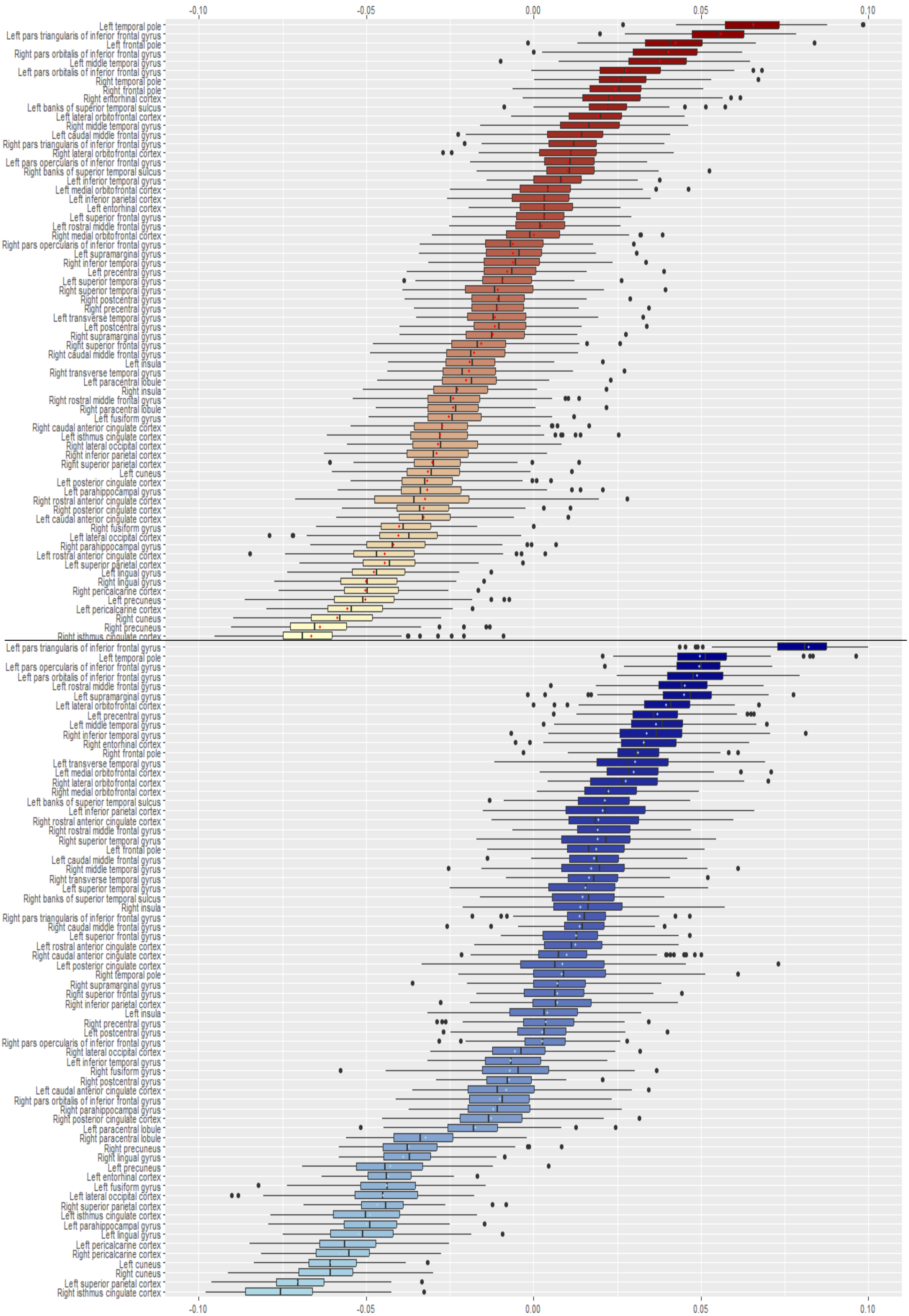

Figure S8. Robustness-Permutation analysis of stability of correlation of epicenter values at the individual level to individual Chlorpromazine analogues. Boxplots represent the distribution of values of the 100 permutations using a different 80% of the sample. Central dot represents the value obtained using 100% of our sample.

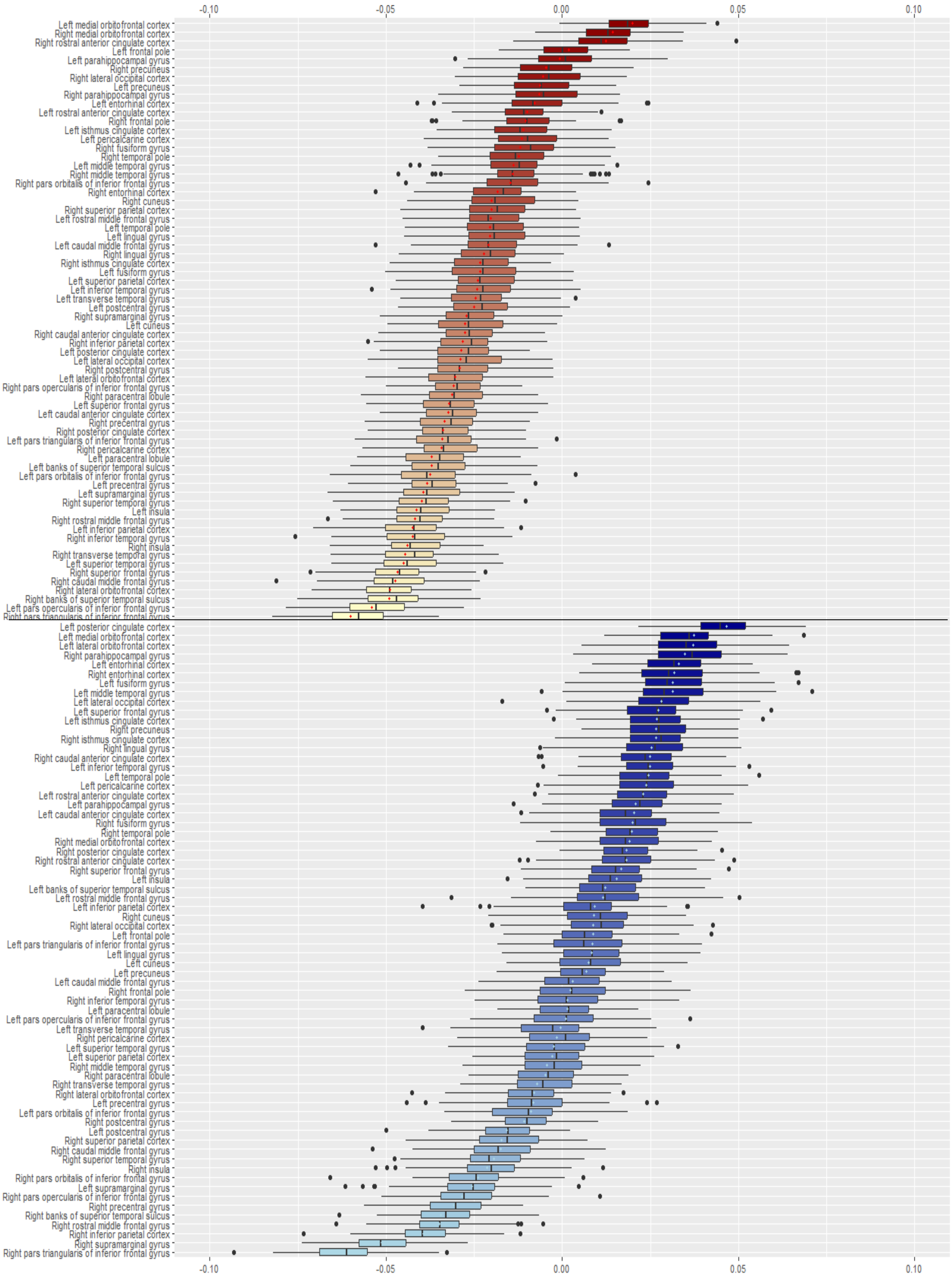

Figure S9. Robustness-Permutation analysis of stability of correlation of epicenter values at the individual level to individual Duration of Illness. Boxplots represent the distribution of values of the 100 permutations using a different 80% of the sample. Central dot represents the value obtained using 100% of our sample.

Cross-modality generalizability of hub vulnerability and epicenter models in surface area alterations of schizophrenia

Most research on cortical hub vulnerability and epicenter models of brain alterations to date has focused on cortical thickness or gray matter volume. Cortical surface area has a heritability of a magnitude similar to cortical thickness, while both are characterized by distinct genetic underpinnings (13, 14). In line with this, the radial hypothesis proposes distinct developmental mechanisms for cortical thickness and surface area. It suggests that cortical thickness results from the neurogenetic division of neural progenitor cells, whereas the expansion of surface area is associated with the propagation of these cells (15). Here we applied our network models to schizophrenia-related cortical surface alterations to test whether the hub vulnerability and epicenters found for cortical thickness alterations are generalizable across both modalities. Thus, these complementary analyses will enable us to identify shared and distinct network mechanisms associated with either one or both cortical measures in schizophrenia. To this end we expanded our original analysis to thoroughly examine the relationship of the normative functional and structural brain networks with surface area alterations in schizophrenia.

Surface area mega-analysis

Using linear models controlling for age and sex across our entire mega-analytic sample (n=2,439 SCZ, n= 2,867 HC), we observed widespread cortical surface area alteration patterns in people with SCZ relative to HC. The derived case-control mega-analytical t-value map closely mirrored the meta-analytic cohen's d effect sizes previously reported by can Erp and colleagues (Fig. S10A; $r = 0.83$, $p_{spin} < 0.0001$) (16). Cortical Surface area reductions in SCZ were in accordance with previous findings (16) and generally lower in mean effect size than CT differences ($MD_{mega}=0.14$, CI = [0.13,0.17]; $t = 15.7$, $df = 67$, $p < 2.2e-16$). However, the spatial pattern of SCZ-related surface area and cortical thickness alteration maps were highly correlated suggesting strong regional agreement of reductions in both modalities. ($r_{mega} = 0.60$, $p_{spin} = 0.0001$). Strongest surface area reductions could be seen in the bilateral fusiform, superior and rostral middle frontal, superior and middle temporal and postcentral gyri (all t-values > 6, FDR $p < 1.57E-07$, Fig. S10A, Table S19). For results of all 68 cortical DKT regions see Table S19.

Functional and structural degree centrality predict regional susceptibility to surface area alterations

Following our cortical thickness analysis, we tested the vulnerability of hubs to surface area reductions in SCZ and compared the spatial patterns of normative functional and structural nodal degree centrality (Fig. 2B) and SCZ-related surface area alterations (Fig. S10B). Mirroring findings from our cortical thickness analyses, a similar vulnerability of cortical hubs could be observed for surface area reductions, in functional ($r = 0.53$, $p_{spin} = 0.0004$) and a trend in the same direction for structural cortico-cortical hubs ($r = 0.22$, $p_{spin}= 0.08$). In sum, functional and structural normative degree centrality predicted the susceptibility of a cortical region to the magnitude of surface area alterations.

Disease epicenters of schizophrenia-related surface area alterations

The epicenter mapping analysis is explained in detail in the main methods section and Figure 3A. In brief, similar to our analysis with SCZ-related cortical thickness alteration, we examined whether the spatial pattern of cortical surface alterations in SCZ are associated with the cortical connection of one or more brain regions to all other cortical regions. A significant relationship between the cortico-cortical connectivity profiles of a region and the pattern of SCZ-related surface alterations suggests that this region could be an epicenter. Such a region might influence the spread of alterations across the cortex in a network-like manner. To identify those regions that might be most likely epicenters, we systematically correlated the normative functional and structural cortico-cortical connectivity profile of each region with the whole-brain patterns of surface area alterations in SCZ (Fig. 3A). Regions were then ranked in descending order based on the strength of their correlation coefficients, with the highest-ranked regions being considered the most significant disease epicenters. The epicenter findings for surface area alterations were very similar to those observed for cortical thickness alterations in SCZ with temporo-paralimbic and frontal regions emerging as the most significant epicenters (Fig. S10C, Table S20). Specifically, the entorhinal cortices, banks of superior temporal sulci, inferior temporal, and additionally frontal gyri (bilateral pars triangularis, left pars opercularis) ranked highest as functional epicenters. For structural epicenters, the right inferior parietal cortex and pars triangularis of the inferior frontal gyrus emerged as significant epicenters of SCZ-related surface area alteration (Fig S10C). In addition, the overall pattern of functional and structural epicenters for surface area alterations correlated highly with the epicenter maps of cortical thickness alteration ($r_{func} = 0.93$, $p_{spin} < 0.0001$, $r_{struc} = 0.85$, $p_{spin} < 0.0001$) confirming the high similarity of epicenters across both cortical measures.

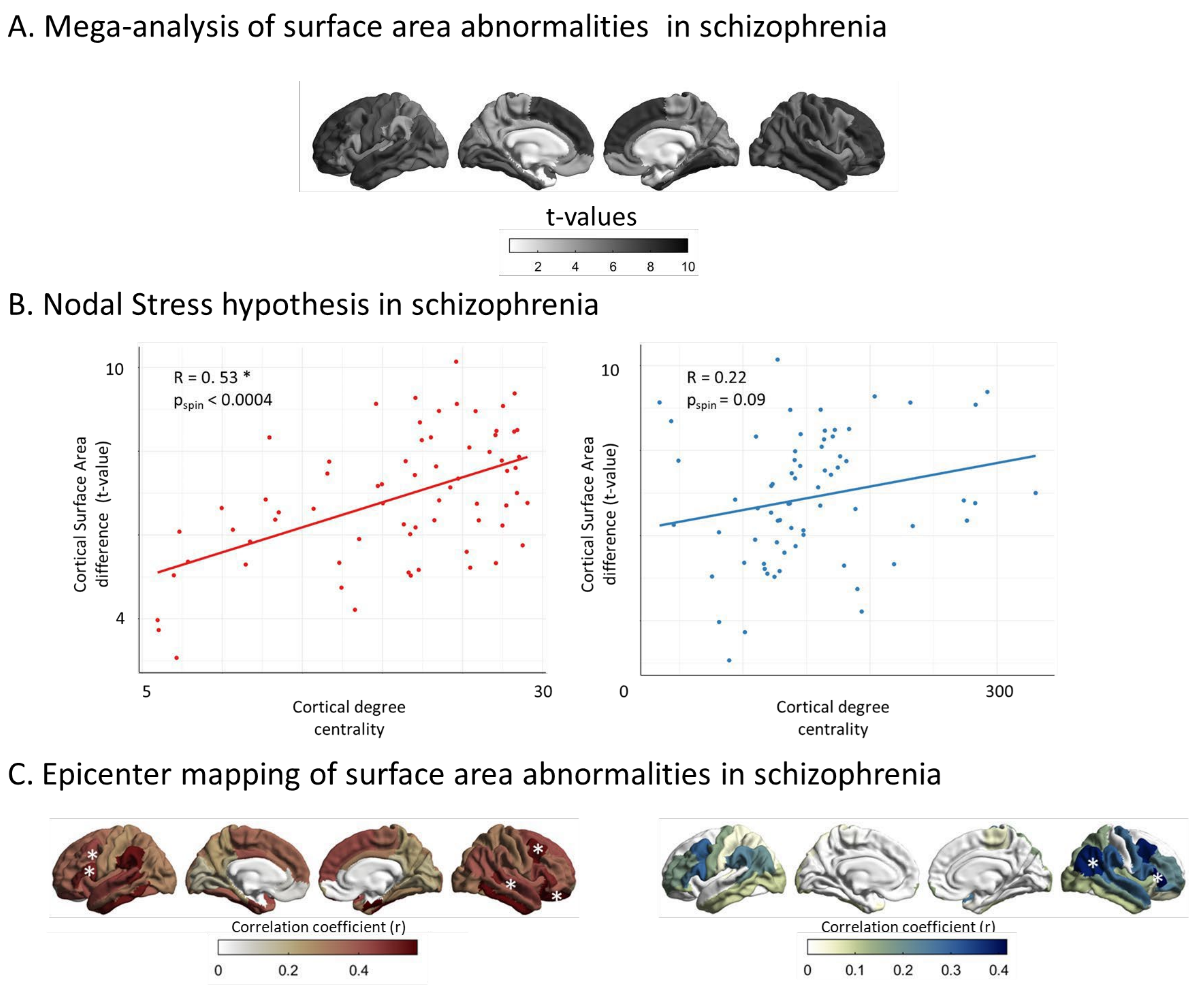

Figure S10. Cross-modality generalizability of mega- and network modelling analysis in surface area. (A) Mega-analytical unthresholded *t*-maps of cortical surface area alterations in SCZ (n = 2,439), compared to HC (n = 2,867). (B) Correlation of cortical surface area alterations with node-level functional (*left*) and structural (*right*) maps of degree centrality. Similar to our cortical thickness findings in SCZ, regions with high functional or structural centrality are significantly more likely to display surface area alterations in the cortex. (C) Correlation coefficient maps depicting the strength of association between the normative region-based functional (*left*) and structural (*right*) connectivity and the SCZ-specific morphological abnormality maps. Disease epicenters are regions more strongly connected with regions showing significant surface area alterations - and, inversely, more weakly connected with regions with less pronounced surface area alterations. Asterisks denote the top five significant epicenters. Top-5 functional epicenters, cortical: (R): lateral orbitofrontal cortex, caudal middle frontal gyrus, middle temporal gyrus (L): caudal middle frontal gyrus, pars opercularis of inferior frontal gyrus. Top-2 structural epicenters: (R): inferior parietal cortex, pars triangularis of inferior frontal gyrus.

Table S19. Surface area differences between schizophrenia patients and healthy controls

Regions	T-values	p values (Bonferroni-corrected)	Cohen's D	Cohen's D 95%CI
Left banks of superior temporal sulcus	8.14	$3.32x10^{-14}$	0.22	[0.17, 0.28]
Left caudal anterior cingulate cortex	7.38	$1.24x10^{-11}$	0.2	[0.15, 0.26]
Left caudal middle frontal gyrus	7.27	$2.71x10^{-11}$	0.2	[0.15, 0.25]
Left cuneus	7.13	$7.77x10^{-11}$	0.2	[0.14, 0.25]
Left entorhinal cortex	7.13	$7.62x10^{-11}$	0.2	[0.14, 0.25]
Left fusiform gyrus	7.08	$1.11x10^{-10}$	0.2	[0.14, 0.25]
Left inferior parietal cortex	6.96	$2.55x10^{-10}$	0.19	[0.14, 0.25]
Left inferior temporal gyrus	6.96	$2.64x10^{-10}$	0.19	[0.14, 0.25]
Left isthmus cingulate cortex	6.69	$1.67x10^{-09}$	0.18	[0.13, 0.24]
Left lateral occipital cortex	6.51	$5.68x10^{-09}$	0.18	[0.13, 0.23]
Left lateral orbitofrontal cortex	6.49	$6.50x10^{-09}$	0.18	[0.12, 0.23]
Left lingual gyrus	6.47	$7.33x10^{-09}$	0.18	[0.12, 0.23]
Left medial orbitofrontal cortex	6.39	$1.25x10^{-08}$	0.18	[0.12, 0.23]
Left middle temporal gyrus	6.33	$1.78x10^{-08}$	0.17	[0.12, 0.23]
Left parahippocampal gyrus	6.33	$1.80x10^{-08}$	0.17	[0.12, 0.23]
Left paracentral lobule	6.26	$2.74x10^{-08}$	0.17	[0.12, 0.23]
Left pars opercularis of inferior frontal gyrus	6.09	$8.22x10^{-08}$	0.17	[0.11, 0.22]
Left pars orbitalis of inferior frontal gyrus	5.98	$1.57x10^{-07}$	0.16	[0.11, 0.22]
Left pars triangularis of inferior frontal gyrus	5.87	$3.22x10^{-07}$	0.16	[0.11, 0.22]
Left pericalcarine cortex	5.78	$5.45x10^{-07}$	0.16	[0.11, 0.21]
Left postcentral gyrus	5.76	$5.94x10^{-07}$	0.16	[0.1, 0.21]
Left posterior cingulate cortex	5.75	$6.34x10^{-07}$	0.16	[0.1, 0.21]
Left precentral gyrus	5.64	$1.23x10^{-06}$	0.16	[0.1, 0.21]
Left precuneus	5.6	$1.52x10^{-06}$	0.15	[0.1, 0.21]
Left rostral anterior cingulate cortex	5.53	$2.25x10^{-06}$	0.15	[0.1, 0.21]
Left rostral middle frontal gyrus	5.47	$3.23x10^{-06}$	0.15	[0.1, 0.2]
Left superior frontal gyrus	5.43	$3.96x10^{-06}$	0.15	[0.1, 0.2]
Left superior parietal cortex	5.35	$6.33x10^{-06}$	0.15	[0.09, 0.2]
Left superior temporal gyrus	5.21	$1.30x10^{-05}$	0.14	[0.09, 0.2]
Left supramarginal gyrus	5.17	$1.63x10^{-05}$	0.14	[0.09, 0.2]
Left frontal pole	5.14	$1.97x10^{-05}$	0.14	[0.09, 0.2]
Left temporal pole	5	$3.97x10^{-05}$	0.14	[0.08, 0.19]
Left transverse temporal gyrus	4.85	$8.77x10^{-05}$	0.13	[0.08, 0.19]

Left insula	4.83	$9.69 x 10^{-05}$	0.13	[0.08, 0.19]
Right banks of superior temporal sulcus	4.76	$1.32 x 10^{-04}$	0.13	[0.08, 0.19]
Right caudal anterior cingulate cortex	4.76	$1.36 x 10^{-04}$	0.13	[0.08, 0.19]
Right caudal middle frontal gyrus	4.75	$1.44 x 10^{-04}$	0.13	[0.08, 0.18]
Right cuneus	4.71	$1.76 x 10^{-04}$	0.13	[0.08, 0.18]
Right entorhinal cortex	4.64	$2.38 x 10^{-04}$	0.13	[0.07, 0.18]
Right fusiform gyrus	4.63	$2.59 x 10^{-04}$	0.13	[0.07, 0.18]
Right inferior parietal cortex	4.54	$3.90 x 10^{-04}$	0.13	[0.07, 0.18]
Right inferior temporal gyrus	4.37	$8.74 x 10^{-04}$	0.12	[0.07, 0.17]
Right isthmus cingulate cortex	4.35	$9.39 x 10^{-04}$	0.12	[0.07, 0.17]
Right lateral occipital cortex	4.35	$9.33 x 10^{-04}$	0.12	[0.07, 0.17]
Right lateral orbitofrontal cortex	4.25	$1.45 x 10^{-03}$	0.12	[0.06, 0.17]
Right lingual gyrus	4.23	$1.64 x 10^{-03}$	0.12	[0.06, 0.17]
Right medial orbitofrontal cortex	4.18	$2.01 x 10^{-03}$	0.12	[0.06, 0.17]
Right middle temporal gyrus	4.13	$2.56 x 10^{-03}$	0.11	[0.06, 0.17]
Right parahippocampal gyrus	4.08	$3.12 x 10^{-03}$	0.11	[0.06, 0.17]
Right paracentral lobule	4.02	$4.02 x 10^{-03}$	0.11	[0.06, 0.16]
Right pars opercularis of inferior frontal gyrus	3.9	$6.51 x 10^{-03}$	0.11	[0.05, 0.16]
Right pars orbitalis of inferior frontal gyrus	3.84	$8.40 x 10^{-03}$	0.11	[0.05, 0.16]
Right pars triangularis of inferior frontal gyrus	3.75	$1.20 x 10^{-02}$	0.1	[0.05, 0.16]
Right pericalcarine cortex	3.6	$2.19 x 10^{-02}$	0.1	[0.05, 0.15]
Right postcentral gyrus	3.36	$5.30 x 10^{-02}$	0.09	[0.04, 0.15]
Right posterior cingulate cortex	3.34	$5.81 x 10^{-02}$	0.09	[0.04, 0.15]
Right precentral gyrus	3.33	$5.98 x 10^{-02}$	0.09	[0.04, 0.15]
Right precuneus	3.29	$6.75 x 10^{-02}$	0.09	[0.04, 0.14]
Right rostral anterior cingulate cortex	3.22	$8.79 x 10^{-02}$	0.09	[0.03, 0.14]
Right rostral middle frontal gyrus	3.17	$1.05 x 10^{-01}$	0.09	[0.03, 0.14]
Right superior frontal gyrus	3.11	$1.30 x 10^{-01}$	0.09	[0.03, 0.14]
Right superior parietal cortex	3.04	$1.63 x 10^{-01}$	0.08	[0.03, 0.14]
Right superior temporal gyrus	3.03	$1.66 x 10^{-01}$	0.08	[0.03, 0.14]
Right supramarginal gyrus	2.74	$4.14 x 10^{-01}$	0.08	[0.02, 0.13]
Right frontal pole	2.22	$1.00 x 10^{+00}$	0.06	[0.01, 0.12]
Right temporal pole	1.97	$1.00 x 10^{+00}$	0.05	[0, 0.11]
Right transverse temporal gyrus	1.73	$1.00 x 10^{+00}$	0.05	[-0.01, 0.1]
Right insula	1.07	$1.00 x 10^{+00}$	0.03	[-0.02, 0.08]

Table S20. Schizophrenia cortical surface area epicenters

Regions	Functional Epicenter R values	Functional Epicenter p_{spin} values (Bonferroni-corrected)	Structural Epicenter R values	Structural Epicenter p_{spin} values (Bonferroni-corrected)
Left banks of superior temporal sulcus	0.5	0.007	0.19	1
Left caudal anterior cingulate cortex	0.34	0.571	-0.04	1
Left caudal middle frontal gyrus	0.42	0	0.29	0.782
Left cuneus	0.16	1	-0.13	1
Left entorhinal cortex	0.45	0.068	-0.2	1
Left fusiform gyrus	0.38	0.095	-0.03	1
Left inferior parietal cortex	0.29	1	0.2	1
Left inferior temporal gyrus	0.51	0.007	0.08	1
Left isthmus cingulate cortex	0.19	1	-0.22	1
Left lateral occipital cortex	0.29	0.585	0.1	1
Left lateral orbitofrontal cortex	0.41	0.027	-0.1	1
Left lingual gyrus	0.15	1	-0.05	1
Left medial orbitofrontal cortex	0.07	1	-0.02	1
Left middle temporal gyrus	0.34	0.197	0.1	1
Left parahippocampal gyrus	0.22	1	-0.01	1
Left paracentral lobule	0.36	0.184	0.01	1
Left pars opercularis of inferior frontal gyrus	0.5	0	0.29	0.456
Left pars orbitalis of inferior frontal gyrus	0.36	0.102	0.11	1
Left pars triangularis of inferior frontal gyrus	0.49	0.007	0.24	1
Left pericalcarine cortex	0.14	1	-0.13	1
Left postcentral gyrus	0.27	1	0.05	1
Left posterior cingulate cortex	0.4	0.177	-0.24	1
Left precentral gyrus	0.3	0.938	0.15	1
Left precuneus	0.24	1	-0.13	1
Left rostral anterior cingulate cortex	0.09	1	-0.16	1
Left rostral middle frontal gyrus	0.35	0.163	0.17	1
Left superior frontal gyrus	0.35	0.272	-0.13	1

Left superior parietal cortex	0.31	0.836	0.04	1
Left superior temporal gyrus	0.43	0.075	-0.04	1
Left supramarginal gyrus	0.53	0.007	0.23	1
Left frontal pole	0.13	1	0.13	1
Left temporal pole	0.37	0.048	0.05	1
Left transverse temporal gyrus	0.45	0.054	0.05	1
Left insula	0.36	0.313	-0.02	1
Right banks of superior temporal sulcus	0.5	0.007	0.27	1
Right caudal anterior cingulate cortex	0.29	1	-0.09	1
Right caudal middle frontal gyrus	0.53	0	0.39	0.068
Right cuneus	0.19	1	-0.1	1
Right entorhinal cortex	0.57	0.007	0.01	1
Right fusiform gyrus	0.27	1	-0.03	1
Right inferior parietal cortex	0.4	0.095	0.38	0.007
Right inferior temporal gyrus	0.54	0.007	0.11	1
Right isthmus cingulate cortex	0.17	1	-0.14	1
Right lateral occipital cortex	0.31	0.415	0.12	1
Right lateral orbitofrontal cortex	0.54	0	0.05	1
Right lingual gyrus	0.19	1	-0.14	1
Right medial orbitofrontal cortex	0.02	1	-0.06	1
Right middle temporal gyrus	0.38	0	0.18	1
Right parahippocampal gyrus	0.27	1	-0.09	1
Right paracentral lobule	0.35	0.374	0.05	1
Right pars opercularis of inferior frontal gyrus	0.43	0.014	0.28	0.422
Right pars orbitalis of inferior frontal gyrus	0.38	0.082	0.24	1
Right pars triangularis of inferior frontal gyrus	0.48	0.014	0.42	0.014
Right pericalcarine cortex	0.22	1	-0.12	1
Right postcentral gyrus	0.29	1	-0.04	1
Right posterior cingulate cortex	0.31	1	-0.15	1
Right precentral gyrus	0.34	0.32	0.27	1
Right precuneus	0.22	1	-0.15	1
Right rostral anterior cingulate cortex	0.05	1	0	1

Right rostral middle frontal gyrus	0.35	0.082	0.21	1
Right superior frontal gyrus	0.41	0.075	-0.15	1
Right superior parietal cortex	0.3	0.707	0.15	1
Right superior temporal gyrus	0.43	0.082	0.26	0.449
Right supramarginal gyrus	0.36	0.197	0.3	0.415
Right frontal pole	0.19	1	0.14	1
Right temporal pole	0.29	0.673	0.02	1
Right transverse temporal gyrus	0.33	0.734	0.11	1
Right insula	0.39	0.163	0.13	1

Subject-level correlations individual hub vulnerability and epicenters of surface area alterations

Subject-level hub vulnerability and clinical variables

Individual R values of cortical hub vulnerability to surface area alterations were, similar to the corresponding hub vulnerability indices for cortical thickness alterations, consistently associated with PANSS general symptom score (r_{func} =0.14, p_{Bonf}=0.033, r_{struc}=0.19, p_{Bonf}=0.001, df = 383) and PANSS total score (r_{func} =0.08, p_{Bonf} =0.065, r_{struc} =0.09, p_{Bonf} =0.026, df = 1039). Robustness of these results were confirmed by running 100 permutations with 80% of the sample each time without resampling (Fig S11, Table S21).

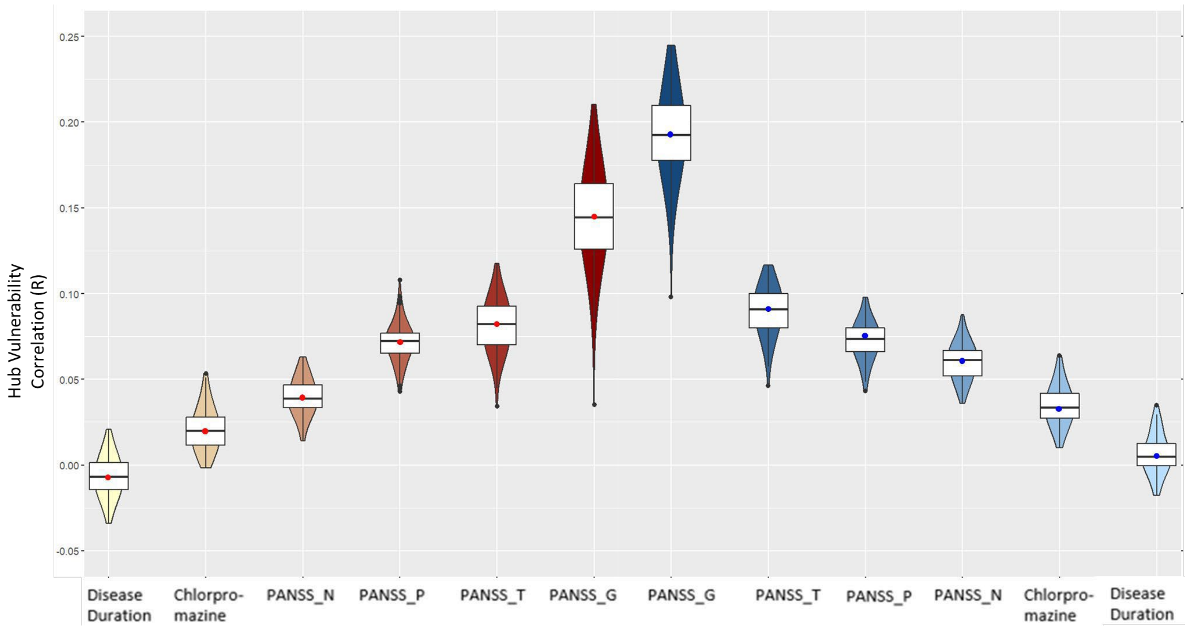

Figure S11. Stability of correlation of hub vulnerability and clinical variables at the individual level. Robustness-Permutation analysis of stability of correlation of hub vulnerability to surface area alterations at the individual level and individual clinical symptoms. Violin plots represent the distribution of values of the 100 permutations using a different 80% of the sample. Central dot represents the value obtained using 100% of our sample.

Table S21. Correlations between subject-level functional and structural hub vulnerability and clinical scores

**Individual Hub Vulnerability**				
	**Functional Connectivity**		**Structural Connectivity**	
Clinical Variables	R	pval (Bonferroni)	R	pval (Bonferroni)
PANSS Positive	0.07	0.027	0.07	0.021
PANSS Negative	0.04	0.586	0.06	0.063
PANSS General	0.14	0.033	0.19	0.001
PANSS Total	0.08	0.065	0.09	0.026
Chlorpromazine	0.02	1	0.03	1
Duration of Illness	-0.01	1	0.01	1
All p-values are corrected for multiple comparison using the Bonferroni method				

Subject-level epicenters and clinical variables

With respect to the correlation between subject-level epicenters and clinical scores, we also found convergent results using individual epicenters cortical thickness and surface area alterations. For both, cortical thickness and surface area, we found significant correlations between the individual epicenter likelihood and higher PANSS general scores (Table S22&S23). Regarding individual functional epicenters, 29 of 68 functional cortical thickness epicenters and 13 surface area epicenters were significantly correlated with higher PANSS general scores (Table S21). 10 of the 13 identified surface area epicenters overlapped with the 29 cortical thickness epicenters suggesting a strong overlap between the association of individual epicenters and symptom scores. To evaluate the statistical significance of the overlap between the sets of significant results between PANSS general symptom scores and higher cortical thickness and surface area epicenter likelihood respectively, we performed a hypergeometric test (N_{total} = 68, N1 = 29, N2 = 13, $N_{overlap}$ = 10; p= 0.007). The overlap of 10 significant results between the two sets is therefore statistically significant, suggesting common neuroanatomical substrates in terms of cortical thickness and surface area regarding their correlation with general symptomatology in schizophrenia. In addition to these significant correlations with functional surface area epicenters, significant correlation between 16 of 68 structural surface area epicenters also correlated significantly with higher PANSS general symptom scores. Together, these findings suggest that individual functional and structural epicenters of surface area are related to higher PANSS general symptomatology.

Table S22. Correlations between individual subject-level functional surface area epicenters and clinical scores

Functional connectivity												
	PANSS Positive		PANSS Negative		PANSS General		PANSS Total		Chlorpromazine equivalents		Duration of Illness	
**Brain Region**	R	pval	R	pval	R	pval	R	pval	R	pval	R	pval
Left banks of superior temporal sulcus	-0.02	1	-0.01	1	-0.07	1	-0.01	1	0.04	1	-0.02	1
Left caudal anterior cingulate cortex	0.09	0.042	0.04	1	0.2	0.008	0.1	0.061	0.02	1	-0.01	1
Left caudal middle frontal gyrus	0.03	1	0.05	1	0.11	1	0.08	0.674	0	1	-0.04	1
Left cuneus	0.02	1	0.02	1	0.05	1	0.01	1	0	1	-0.03	1
Left entorhinal cortex	0.02	1	-0.01	1	0.05	1	0.02	1	0	1	-0.03	1
Left fusiform gyrus	0.01	1	0.04	1	0.01	1	0.03	1	-0.01	1	-0.03	1
Left inferior parietal cortex	0	1	-0.01	1	-0.01	1	0	1	0.02	1	-0.02	1
Left inferior temporal gyrus	0.03	1	0.03	1	0.05	1	0.03	1	0.05	1	-0.03	1
Left isthmus cingulate cortex	0.06	0.727	-0.01	1	0.1	1	0.05	1	0.01	1	-0.03	1
Left lateral occipital cortex	0.06	1	0.08	0.018	0.05	1	0.09	0.332	0	1	-0.03	1
Left lateral orbitofrontal cortex	0.03	1	0.03	1	0.22	0.001	0.04	1	0.03	1	-0.01	1
Left lingual gyrus	0.01	1	0.02	1	0.05	1	0.04	1	0.05	1	-0.03	1
Left medial orbitofrontal cortex	0.02	1	0.05	1	0.12	0.72	0.06	1	0	1	-0.03	1
Left middle temporal gyrus	0.03	1	0.01	1	0.05	1	0.04	1	0.03	1	-0.02	1
Left parahippocampal gyrus	0.03	1	0	1	0.09	1	0.03	1	0.02	1	0	1

Left paracentral lobule	0.03	1	0.03	1	0.15	0.174	0.05	1	-0.01	1	-0.02	1
Left pars opercularis of inferior frontal gyrus	0.03	1	0.01	1	0.07	1	0.04	1	0.02	1	0.01	1
Left pars orbitalis of inferior frontal gyrus	0.04	1	0.05	1	0.09	1	0.05	1	-0.05	1	0	1
Left pars triangularis of inferior frontal gyrus	0.03	1	0.02	1	0.15	0.197	0.05	1	0.02	1	-0.03	1
Left pericalcarine cortex	0.02	1	0.02	1	0.02	1	0.03	1	0.02	1	-0.04	1
Left postcentral gyrus	0.05	1	0.06	0.544	0.18	0.017	0.11	0.044	0.02	1	-0.01	1
Left posterior cingulate cortex	0.07	0.162	0.04	1	0.18	0.02	0.09	0.175	0.02	1	-0.04	1
Left precentral gyrus	0.08	0.057	0.07	0.101	0.2	0.005	0.11	0.017	0.04	1	-0.02	1
Left precuneus	0.03	1	0.03	1	0.06	1	0.05	1	0.01	1	0	1
Left rostral anterior cingulate cortex	0.05	1	0.04	1	0.17	0.061	0.07	1	0.02	1	-0.02	1
Left rostral middle frontal gyrus	0.02	1	0.02	1	0.01	1	0.02	1	0.04	1	-0.01	1
Left superior frontal gyrus	0.03	1	0.06	0.927	0.13	0.609	0.07	0.869	0.02	1	-0.04	1
Left superior parietal cortex	0.1	0.003	0.08	0.039	0.23	0	0.15	0	0	1	-0.05	1
Left superior temporal gyrus	0.04	1	0	1	0.05	1	0.03	1	0.01	1	-0.06	0.495
Left supramarginal gyrus	0.04	1	0	1	0.05	1	0.04	1	-0.01	1	-0.05	1
Left frontal pole	0.02	1	0.04	1	0.13	0.397	0.06	1	-0.01	1	0.01	1
Left temporal pole	-0.03	1	-0.01	1	-0.02	1	-0.02	1	0	1	-0.01	1
Left transverse temporal gyrus	0.02	1	0.02	1	0.13	0.422	0.07	1	0.04	1	-0.04	1

Left insula	0	1	0.01	1	0.13	0.534	0.01	1	0.03	1	-0.04	1
Right banks of superior temporal sulcus	0	1	-0.02	1	-0.04	1	-0.02	1	0.01	1	-0.05	1
Right caudal anterior cingulate cortex	0.05	1	0.02	1	0.1	1	0.06	1	0.04	1	-0.06	0.47
Right caudal middle frontal gyrus	0.08	0.063	0.06	0.613	0.14	0.352	0.11	0.026	0.01	1	-0.01	1
Right cuneus	0.03	1	0.01	1	0.1	1	0.03	1	-0.02	1	-0.03	1
Right entorhinal cortex	0.01	1	-0.01	1	0.03	1	0.01	1	-0.01	1	0	1
Right fusiform gyrus	-0.02	1	-0.01	1	0.05	1	0.02	1	0	1	-0.03	1
Right inferior parietal cortex	0.02	1	0.01	1	0.07	1	0.03	1	0.01	1	-0.01	1
Right inferior temporal gyrus	0.04	1	0.03	1	0.06	1	0.04	1	0.01	1	-0.01	1
Right isthmus cingulate cortex	0.05	1	0.04	1	0.18	0.017	0.06	1	0.02	1	-0.06	0.316
Right lateral occipital cortex	0.03	1	0.04	1	0.02	1	0.05	1	0.14	0	-0.03	1
Right lateral orbitofrontal cortex	0.02	1	0.01	1	0.09	1	0.02	1	0.02	1	0	1
Right lingual gyrus	0.02	1	0.04	1	0.02	1	0.05	1	0.02	1	-0.05	1
Right medial orbitofrontal cortex	0.03	1	0.04	1	0.11	1	0.06	1	0.01	1	-0.02	1
Right middle temporal gyrus	0.02	1	0.01	1	0.03	1	0.02	1	0.03	1	-0.03	1
Right parahippocampal gyrus	-0.01	1	-0.05	1	0.01	1	-0.03	1	0.05	1	-0.02	1
Right paracentral lobule	0.07	0.238	0.04	1	0.19	0.01	0.1	0.085	0	1	-0.02	1

Right pars opercularis of inferior frontal gyrus	0.03	1	0.05	1	0.18	0.025	0.06	1	0.03	1	0	1
Right pars orbitalis of inferior frontal gyrus	0.06	0.968	0.02	1	0.01	1	0.04	1	0.05	1	0.01	1
Right pars triangularis of inferior frontal gyrus	0.03	1	0.04	1	0.07	1	0.05	1	0	1	-0.04	1
Right pericalcarine cortex	0.02	1	0.02	1	0.03	1	0.03	1	0.02	1	-0.02	1
Right postcentral gyrus	0.08	0.093	0.05	1	0.18	0.024	0.12	0.012	0.01	1	-0.02	1
Right posterior cingulate cortex	0.06	0.675	0.03	1	0.19	0.011	0.09	0.234	0.03	1	-0.02	1
Right precentral gyrus	0.08	0.054	0.07	0.087	0.11	1	0.1	0.069	0.02	1	-0.02	1
Right precuneus	0.04	1	0.03	1	0.15	0.205	0.05	1	0.01	1	-0.02	1
Right rostral anterior cingulate cortex	0.03	1	0.01	1	0.15	0.214	0.05	1	0	1	-0.04	1
Right rostral middle frontal gyrus	0.01	1	0.02	1	0.06	1	0.05	1	0.05	1	-0.04	1
Right superior frontal gyrus	0.07	0.503	0.06	0.446	0.18	0.022	0.1	0.061	0.03	1	-0.02	1
Right superior parietal cortex	0.06	1	0.06	0.339	0.19	0.012	0.09	0.175	0.03	1	-0.02	1
Right superior temporal gyrus	0.04	1	0.02	1	0.07	1	0.04	1	0.01	1	-0.07	0.113
Right supramarginal gyrus	-0.01	1	0	1	0	1	-0.01	1	0.01	1	-0.01	1
Right frontal pole	0.02	1	0.04	1	0.11	1	0.05	1	0.02	1	0	1
Right temporal pole	0.01	1	0.03	1	0.04	1	0.02	1	-0.02	1	-0.01	1
Right transverse temporal gyrus	0.02	1	0.03	1	0.12	1	0.04	1	0.01	1	-0.03	1

Right insula	0.05	1	0.04	1	0.13	0.593	0.07	1	0.05	1	-0.06	0.257
All p-values are corrected for multiple comparison using the Bonferroni method												

Table S23. Correlations between individual subject-level structural surface area epicenters and clinical scores

**Structural connectivity**												
	PANSS Positive		PANSS Negative		PANSS General		PANSS Total		Chlorpromazine equivalents		Duration of Illness	
**Brain Region**	R	pval	R	pval	R	pval	R	pval	R	pval	R	pval
Left banks of superior temporal sulcus	0.03	1	-0.05	1	-0.03	1	-0.02	1	-0.02	1	-0.01	1
Left caudal anterior cingulate cortex	0.03	1	0.04	1	0.12	0.765	0.05	1	0.02	1	0.03	1
Left caudal middle frontal gyrus	0.08	0.136	0.06	0.774	0.2	0.006	0.1	0.107	0.02	1	0.02	1
Left cuneus	0.02	1	0.03	1	0.01	1	0.03	1	0.03	1	-0.03	1
Left entorhinal cortex	-0.07	0.224	-0.05	1	-0.22	0.001	-0.11	0.042	-0.02	1	-0.01	1
Left fusiform gyrus	0.01	1	-0.01	1	-0.06	1	-0.02	1	0.03	1	-0.03	1
Left inferior parietal cortex	0.06	1	0.04	1	0.1	1	0.05	1	-0.01	1	-0.01	1
Left inferior temporal gyrus	0	1	-0.01	1	-0.05	1	-0.03	1	-0.02	1	-0.02	1
Left isthmus cingulate cortex	0.01	1	0.01	1	0.04	1	0.01	1	-0.01	1	0	1
Left lateral occipital cortex	-0.01	1	-0.03	1	-0.07	1	-0.05	1	-0.01	1	0	1
Left lateral orbitofrontal cortex	-0.01	1	0	1	0	1	-0.01	1	0	1	-0.02	1
Left lingual gyrus	0	1	0.01	1	-0.05	1	-0.01	1	-0.02	1	-0.03	1
Left medial orbitofrontal cortex	0.01	1	0.03	1	0.04	1	0.01	1	0.04	1	0.02	1

Left middle temporal gyrus	0.01	1	-0.01	1	-0.03	1	-0.02	1	-0.01	1	-0.02	1
Left parahippocampal gyrus	0.01	1	0.03	1	0.01	1	0.02	1	0	1	0.01	1
Left paracentral lobule	0.09	0.024	0.09	0.014	0.25	0	0.13	0.001	0.01	1	0.03	1
Left pars opercularis of inferior frontal gyrus	0.06	1	0.03	1	0.14	0.359	0.06	1	0.01	1	0	1
Left pars orbitalis of inferior frontal gyrus	0.03	1	0.01	1	0.06	1	0.01	1	0.01	1	-0.02	1
Left pars triangularis of inferior frontal gyrus	0.05	1	0.03	1	0.1	1	0.04	1	0.03	1	0	1
Left pericalcarine cortex	0	1	0.01	1	-0.03	1	0	1	-0.02	1	-0.02	1
Left postcentral gyrus	0.07	0.421	0.02	1	0.18	0.025	0.08	0.567	-0.02	1	0	1
Left posterior cingulate cortex	0.05	1	0.06	0.661	0.17	0.054	0.08	0.504	0.01	1	0.04	1
Left precentral gyrus	0.07	0.264	0.05	1	0.21	0.002	0.1	0.049	0	1	0.02	1
Left precuneus	0.07	0.296	0.05	1	0.2	0.004	0.09	0.17	0.03	1	0	1
Left rostral anterior cingulate cortex	0	1	0.04	1	0.08	1	0.03	1	0	1	0.03	1
Left rostral middle frontal gyrus	0.04	1	0.06	0.362	0.16	0.087	0.07	0.951	0.01	1	0.04	1
Left superior frontal gyrus	0.04	1	0.05	1	0.19	0.007	0.08	0.402	0.02	1	0.03	1
Left superior parietal cortex	0.04	1	0.01	1	0.03	1	0.03	1	0	1	-0.02	1
Left superior temporal gyrus	0	1	-0.01	1	0.01	1	0	1	-0.04	1	-0.01	1

Left supramarginal gyrus	0.03	1	0	1	0.11	1	0.02	1	-0.01	1	0.01	1
Left frontal pole	0.04	1	0.06	0.692	0.14	0.304	0.07	1	0.03	1	0.01	1
Left temporal pole	-0.02	1	0	1	-0.06	1	-0.05	1	-0.03	1	-0.03	1
Left transverse temporal gyrus	0.04	1	-0.02	1	0.09	1	0.04	1	-0.02	1	-0.04	1
Left insula	0.06	0.962	0.05	1	0.15	0.197	0.06	1	-0.01	1	0.01	1
Right banks of superior temporal sulcus	0.01	1	-0.04	1	-0.03	1	-0.03	1	-0.01	1	0.01	1
Right caudal anterior cingulate cortex	0.04	1	0.06	0.94	0.18	0.022	0.08	0.711	0.03	1	0.02	1
Right caudal middle frontal gyrus	0.07	0.488	0.06	0.55	0.2	0.006	0.09	0.222	0.03	1	0.01	1
Right cuneus	0.02	1	0.02	1	0	1	0.02	1	0.04	1	-0.02	1
Right entorhinal cortex	-0.01	1	0	1	-0.02	1	-0.02	1	-0.01	1	0	1
Right fusiform gyrus	0.01	1	0	1	-0.05	1	0	1	0.08	0.307	-0.03	1
Right inferior parietal cortex	0.07	0.269	0.05	1	0.16	0.089	0.09	0.212	0.05	1	0.01	1
Right inferior temporal gyrus	0.01	1	-0.01	1	-0.02	1	0	1	0.04	1	-0.02	1
Right isthmus cingulate cortex	0.01	1	0.01	1	0.01	1	0.02	1	0.03	1	-0.01	1
Right lateral occipital cortex	0	1	-0.02	1	-0.05	1	-0.03	1	0	1	-0.02	1
Right lateral orbitofrontal cortex	0.01	1	0.04	1	-0.01	1	0.02	1	0.07	0.488	-0.03	1
Right lingual gyrus	-0.02	1	-0.01	1	-0.08	1	-0.02	1	0.04	1	-0.01	1
Right medial orbitofrontal cortex	0.02	1	0.03	1	0.1	1	0.03	1	0.03	1	0.03	1
Right middle temporal gyrus	0	1	-0.01	1	-0.07	1	-0.03	1	0.05	1	-0.02	1

Right parahippocampal gyrus	0	1	-0.01	1	-0.05	1	-0.01	1	0.04	1	-0.01	1
Right paracentral lobule	0.08	0.136	0.08	0.044	0.23	0	0.12	0.013	0	1	0.03	1
Right pars opercularis of inferior frontal gyrus	0.07	0.299	0.06	0.701	0.2	0.003	0.1	0.06	0.02	1	0.02	1
Right pars orbitalis of inferior frontal gyrus	0.02	1	0.01	1	0.01	1	0	1	0.06	1	-0.01	1
Right pars triangularis of inferior frontal gyrus	0.06	0.992	0.03	1	0.13	0.469	0.06	1	0.03	1	0.02	1
Right pericalcarine cortex	0.03	1	0.03	1	0.01	1	0.03	1	0.04	1	-0.04	1
Right postcentral gyrus	0.08	0.098	0.06	0.613	0.22	0.001	0.11	0.04	0	1	0.02	1
Right posterior cingulate cortex	0.07	0.204	0.07	0.092	0.22	0.001	0.11	0.016	0.01	1	0.04	1
Right precentral gyrus	0.07	0.18	0.06	0.618	0.24	0	0.11	0.029	0.02	1	0.02	1
Right precuneus	0.02	1	-0.01	1	0.06	1	0.03	1	0.01	1	0	1
Right rostral anterior cingulate cortex	0.04	1	0.05	1	0.14	0.284	0.06	1	0.02	1	0.03	1
Right rostral middle frontal gyrus	0.06	0.962	0.06	0.445	0.18	0.022	0.09	0.332	0.05	1	0.02	1
Right superior frontal gyrus	0.04	1	0.07	0.255	0.2	0.003	0.08	0.448	0.03	1	0.02	1
Right superior parietal cortex	0.06	0.903	0.02	1	0.1	1	0.07	1	0.02	1	-0.01	1
Right superior temporal gyrus	0	1	-0.01	1	-0.05	1	-0.04	1	0.05	1	0.01	1
Right supramarginal gyrus	0.04	1	0.02	1	0.12	0.966	0.04	1	0	1	0.01	1

Right frontal pole	0.04	1	0.05	1	0.11	1	0.05	1	0.03	1	0	1
Right temporal pole	-0.04	1	-0.02	1	-0.15	0.186	-0.07	1	0.04	1	-0.01	1
Right transverse temporal gyrus	0.03	1	0.01	1	0.05	1	0.01	1	0	1	-0.01	1
Right insula	0.04	1	0.05	1	0.13	0.469	0.08	0.704	0.04	1	0.01	1
All p-values are corrected for multiple comparison using the Bonferroni method												

Cross-disorder comparison of hub vulnerability and epicenter mapping

Similar to our cortical thickness analysis, we further probed whether our hub and epicenter findings for surface area alterations in SCZ are specific or represent associations that are shared across the schizophrenia-affective disorder spectrum. To this end, we leveraged the meta-analytic cortical surface area case-control maps of bipolar disorder (BD) and major depressive disorder (MDD) (17, 18). Convergent with our cortical thickness findings, surface area alterations in BD were moderately correlated with cortico-cortical hubs ($r_{func} = 0.3$, $p_{spin} = 0.054$; $r_{struc} = 0.2$, $p_{spin} = 0.15$) while no associations were found in MDD (all $p_{spin} > 0.05$) (Fig. S12A). These findings confirm our hub vulnerability models of cortical thickness alterations showing significant hub vulnerability for surface area alterations in SCZ and BD but not MDD. In accordance with our cortical thickness analysis, the left caudal middle frontal gyrus emerged as significant disease epicenter of surface area alterations in BD, whereas no significant epicenters were observed for surface are alterations in MDD (Fig. S12B). These findings mirror our original cross-disorder comparison with cortical thickness alterations showing that the magnitude of observed cortical disease epicenters was most pronounced in SCZ, intermediate in BD and relatively lacking in MDD.

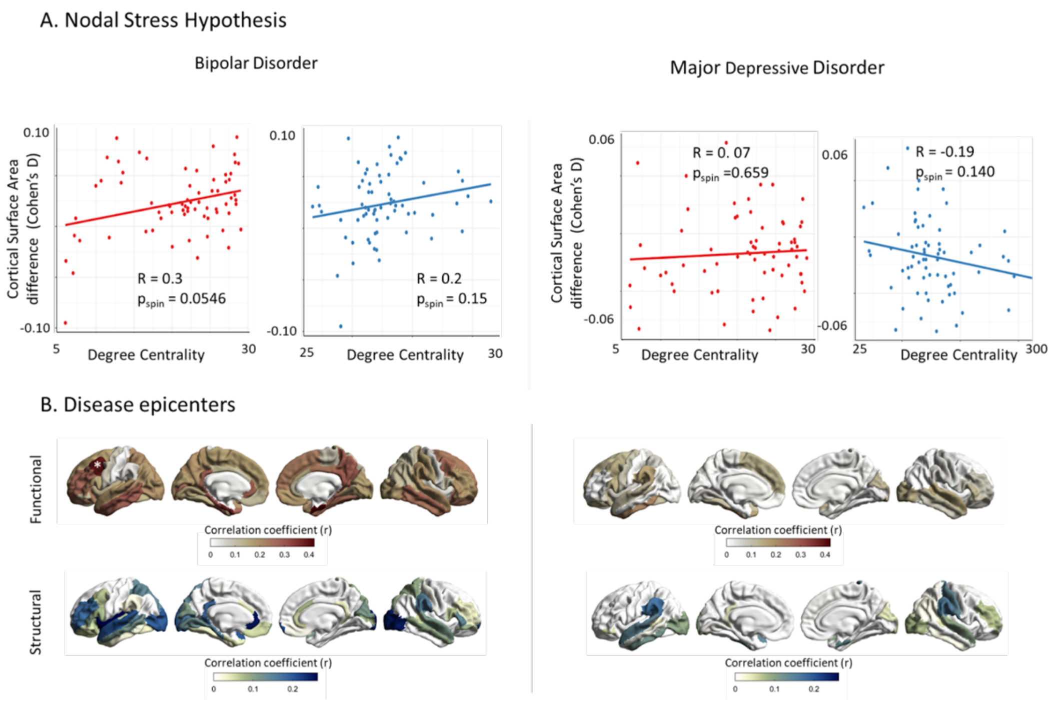

Figure S12. Cross-disorder comparison of network modeling of cortical surface area alterations. (A) Correlation of disorder-related surface area alterations to node-level functional (left) and structural (right) maps of degree centrality in BD and MDD. Convergent with our cortical thickness analysis, in BD, cortical regions with high structural centrality are significantly more likely to display higher surface area alterations. No such relationship is observed in MDD. (B) Correlation coefficient maps depicting strength of association between each region normative functional (top) and structural (bottom) connectivity and the BD-specific surface area alteration map (left) and the MDD-specific surface area alteration map (right). Asterisks denote the top five significant epicenters. Functional epicenters in BD: Left caudal middle frontal gyrus. No significant epicenters could be detected in MDD.

Acknowledgments per dataset

Acknowledgments per dataset are as follows:

ASRB: The Australian Schizophrenia Research Bank (ASRB), was supported by the National Health and Medical Research Council of Australia (NHMRC) (Enabling Grant, ID 386500), the Pratt Foundation, Ramsay Health Care, the Viertel Charitable Foundation and the Schizophrenia Research Institute. Chief Investigators for ASRB were Carr, V., Schall, U., Scott, R., Jablensky, A., Mowry, B., Michie, P., Catts, S., Henskens, F., Pantelis, C. We thank Loughland, C., the ASRB Manager, and acknowledge the help of Jason Bridge with ASRB database queries. CP was supported by NHMRC Senior Principal Research Fellowships (IDs: 628386 & 1105825); GC was supported by the Schizophrenia Research Institute utilizing infrastructure funding from the New South Wales Ministry of Health and New South Wales Ministry of Trade and Investment (Australia); JF was supported by NHMRC project grant (1063960); MG was supported by NHMRC as an R.D. Wright Biomedical Career Development Fellow (1061875). MJC was supported by NHMRC Senior Research Fellowship (1121474). CSW is funded by the NSW Ministry of Health, Office of Health and Medical Research. CSW is a recipient of a National Health and Medical Research Council (Australia) Principal Research Fellowship (PRF) (#1117079).

CAMH: The CAMH datasets were collected and shared with support from the CAMH Foundation and the Canadian Institutes of Health Research.

CIAM: The CIAM study is supported by the South African Medical Research Council and National Research Foundation of South Africa.

COBRE: The COBRE dataset and investigators were supported by NIH grants R01EB006841 & P20GM103472, as well as NSF grant 1539067. JT (senior author) and VDC are supported by 5R01MH094524. JMS is supported by R01 AA021771 and P50 AA022534.

ESO: The ESO study was funded by NPU I – LO1611 and Ministry of Health, Czech Republic – Conceptual Development of Research Organization 00023001 (IKEM).

FIDMAG: Supported by Instituto de Salud Carlos III (Co-funded by European Regional Development Fund/European Social Fund) "Investing in your future"): Miguel Servet Research Contract (CPII16/00018 to E. Pomarol-Clotet and CP14/00041 to J. Radua.).

FOR2107 Marburg: This work was funded by the German Research Foundation (DFG), Tilo Kircher (speaker FOR2107; DFG grant numbers KI 588/14-1, KI 588/14-2), Axel Krug (KR 3822/5-1, KR 3822/7-2), Igor Nenadic (NE 2254/1-2), Carsten Konrad (KO 4291/3-1).

FOR2107 Muenster: The FOR2107 Muenster study was funded by the German Research Foundation (DFG, grant FOR2107 DA1151/5-1 and DA1151/5-2 to UD) and the Interdisciplinary Center for Clinical Research (IZKF) of the medical faculty of Münster (grant Dan3/012/17 to UD).

FSL_Rome: This study was supported by grants (RC10-11-12-13-14-15/A) from the Italian Ministry of Health and by the ERANET NEURON from the European Commission.

MPRC: Support was received from NIH grants U01MH108148, 2R01EB015611, R01MH112180, R01DA027680, R01MH085646, P50MH103222 and T32MH067533, a State of Maryland contract (M00B6400091) and NSF grant (1620457).

OLIN: The Olin study was supported by NIH grants R37MH43375 and R01MH074797.

PAFIP: The PAFIP study was supported by Instituto de Salud Carlos III, MINECOSAF2013-46292-R, PSYSCAN (Exp.: HEALTH.2013.2.2.1-2_Grant agreement no. 603196), FIS PI14/00639. We want to particularly acknowledge the patients and the BioBankValdecilla

(PT13/0010/0024) integrated in the Spanish National Biobanks Network for its collaboration. We thank IDIVAL Neuroimaging Unit for its help in the technical execution of this work.

PENS: This work was supported by the awards by the Department of Veterans Affairs Clinical Science Research and Development Service (Grant No. I01CX000227 [to SRS]) and the National Institute of Mental Health of the National Institutes of Health (NIH) (Grant No. R01MH112583 [to SRS]). Thiswork is also supported by the National Institute of Neurological Disorders and Stroke of the NIH (Grant No. P30 NS076408), the National Eye Institute of the NIH (Grant No. P30 EY011374), the National Institute of Biomedical Imaging and Bioengineering of the NIH (Grant No. P41EB015894), and the NIH (Grant No. 1S10OD017974-01). The content is solely the responsibility of the authors and does not necessarily represent the official views of the Department of Veterans Affairs or the NIH.

PHCP: This work was supported by the National Institutes of Health (U01MH108150) and the Center for Magnetic Resonance Research (P41 EB027061; 1S10OD017974).

SCORE: This study was supported in part by grant 3232BO_119382 from the Swiss National Science Foundation. We thank the FePsy (Frueherkennung von Psychosen; early detection of psychosis) Study Group from the University of Basel, Department of Psychiatry, Switzerland, for the recruitment of the study participants. The FePsy Study was supported in part by grant No. SNF 3200^{-05}7216/1, ext./2, ext./3.

Singapore: This work was supported by research grants from the National Healthcare Group, Singapore, and the Singapore Bioimaging Consortium research grants awarded to K.S.

SWIFT: This research was supported in part by the Swiss National Science Foundation (grant no. 320030_146789).

UCISZ: The UCISZ study was supported by the National Institutes of Mental Health grant number R21MH097196 to TGMvE. UCISZ data was processed by the UCI High Performance Computing cluster supported by Joseph Farran, Harry Mangalam, and Adam Brenner and the National Center for Research Resources and the National Center for Advancing Translational Sciences, National Institutes of Health, through Grant UL1 TR000153. AB was also supported by NIH grants 5R01 MH61603, and 2R01MH058251; JF by NIMH (R01 MH-58262).

UPENN: The UPENN study was supported by National Institute of Mental Health grants MH064045, MH60722, MH019112, MH085096 (DHW), and R01MH10770 (TDS).

Zurich: This study was supported by the Swiss National Science Foundation (Grant No. 105314_140351 to SK, Grant No. P2SKP3_178175 to MK).

References

1. Van Essen DC, Ugurbil K, Auerbach E, Barch D, Behrens TEJ, Bucholz R, *et al.* The Human Connectome Project: A data acquisition perspective. *Neuroimage* **62**, 2222 (2012).

2. Glasser MF, Sotiropoulos SN, Wilson JA, Coalson TS, Fischl B, Andersson JL, *et al.* The Minimal Preprocessing Pipelines for the Human Connectome Project. *Neuroimage* **80**, 105 (2013).

3. Desikan RS, Ségonne F, Fischl B, Quinn BT, Dickerson BC, Blacker D, *et al.* An automated labeling system for subdividing the human cerebral cortex on MRI scans into gyral based regions of interest. *Neuroimage* **31**, 968–980 (2006).

4. Smith RE, Tournier JD, Calamante F, Connelly A. Anatomically-constrained tractography: Improved diffusion MRI streamlines tractography through effective use of anatomical information. *Neuroimage* **62**, 1924–1938 (2012).

5. Jeurissen B, Tournier JD, Dhollander T, Connelly A, Sijbers J. Multi-tissue constrained spherical deconvolution for improved analysis of multi-shell diffusion MRI data. *Neuroimage* **103**, 411–426 (2014).

6. Smith RE, Tournier JD, Calamante F, Connelly A. SIFT2: Enabling dense quantitative assessment of brain white matter connectivity using streamlines tractography. *Neuroimage* **119**, 338–351 (2015).

7. Tournier JD, Calamante F, Connelly A. Robust determination of the fibre orientation distribution in diffusion MRI: Non-negativity constrained super-resolved spherical deconvolution. *Neuroimage* **35**, 1459–1472 (2007).

8. Desikan RS, Ségonne F, Fischl B, Quinn BT, Dickerson BC, Blacker D, *et al.* An automated labeling system for subdividing the human cerebral cortex on MRI scans into gyral based regions of interest. *Neuroimage* **31**, 968–980 (2006).

9. Tournier JD, Smith R, Raffelt D, Tabbara R, Dhollander T, Pietsch M, *et al.* MRtrix3: A fast, flexible and open software framework for medical image processing and visualisation. *Neuroimage* **202**, 116137 (2019).

10. Betzel RF, Griffa A, Hagmann P, Mišić B. Distance-dependent consensus thresholds for generating group-representative structural brain networks. *Network Neuroscience* **3**, 475–496 (2019).

11. Heuvel MP van den, Sporns O, Collin G, Scheewe T, Mandl RCW, Cahn W, *et al.* Abnormal Rich Club Organization and Functional Brain Dynamics in Schizophrenia. *JAMA Psychiatry* **70**, 783–792 (2013).

12. Zuo XN, Ehmke R, Mennes M, Imperati D, Castellanos FX, Sporns O, *et al.* Network Centrality in the Human Functional Connectome. *Cerebral Cortex* **22**, 1862–1875 (2012).

13. Grasby KL, Jahanshad N, Painter JN, Colodro-Conde L, Bralten J, Hibar DP, *et al.* The genetic architecture of the human cerebral cortex. *Science (1979)* **367**, (2020).

14. Grotzinger AD, Mallard TT, Liu Z, Seidlitz J, Ge T, Smoller JW. Multivariate genomic architecture of cortical thickness and surface area at multiple levels of analysis. *Nature Communications 2023 14:1* **14**, 1–13 (2023).

15. Rakic P. Specification of Cerebral Cortical Areas. *Science (1979)* **241**, 170–176 (1988).

16. van Erp TGM, Walton E, Hibar DP, Schmaal L, Jiang W, Glahn DC, *et al.* Cortical Brain Abnormalities in 4474 Individuals With Schizophrenia and 5098 Control Subjects via the Enhancing Neuro Imaging Genetics Through Meta Analysis (ENIGMA) Consortium. *Biol Psychiatry* **84**, 644–654 (2018).

17. Schmaal L, Hibar DP, Sämann PG, Hall GB, Baune BT, Jahanshad N, *et al.* Cortical abnormalities in adults and adolescents with major depression based on brain scans from 20 cohorts worldwide in the ENIGMA Major Depressive Disorder Working Group. *Mol Psychiatry* **22**, 900–909 (2017).

18. Hibar DP, Westlye LT, Doan NT, Jahanshad N, Cheung JW, Ching CRK, *et al.* Cortical abnormalities in bipolar disorder: An MRI analysis of 6503 individuals from the ENIGMA Bipolar Disorder Working Group. *Mol Psychiatry* **23**, 932–942 (2018).
